# Distinct roles of clustered MicroRNAs miR-286 and miR-6 in JNK activation critical to apoptosis-induced proliferation in *Drosophila*

**DOI:** 10.1007/s00018-025-05880-w

**Published:** 2025-11-25

**Authors:** Mengyuan Yu, Caitlin Hounsell, Buyun Zhang, Tingxuan Wang, Xiaolin Bi, Yun Fan

**Affiliations:** 1 School of Biosciences, Birmingham Centre for Neurogenetics, University of Birmingham, Birmingham, UK; 2 School of Medicine, Nantong University, Nantong, China; 3 Division of Life Science and State Key Laboratory of Molecular Neuroscience, The Hong Kong University of Science and Technology, Hongkong, China

**Keywords:** MicroRNA cluster, JNK, Apoptosis-induced proliferation, Calcium signaling, *Drosophila*

## Abstract

Apoptosis-induced proliferation (AiP) is an evolutionarily conserved process implicated in tissue regeneration and tumorigenesis. Studies in *Drosophila* have identified activation of the stress response molecule c-Jun N-terminal kinase (JNK) as a critical step in mediating AiP. Interestingly, JNK activation can be further amplified to drive tissue overgrowth during this process. However, the mechanisms that coordinate the initial activation of JNK and its subsequent amplification remain poorly understood. In this study, we identified distinct functions for two members of the microRNA cluster miR-309/3/286/4/5/6 − 1/6 − 2/6 − 3, specifically miR-286 and miR-6, in regulating JNK signaling during AiP. We found that miR-6 promoted the initial activation of JNK, whereas miR-286 inhibited its amplification. During AiP, the expression of miR-286 was reduced, and we identified *Calx*, a gene encoding a sodium/calcium exchanger involved in intracellular calcium homeostasis, as a direct target of miR-286. Loss of miR-286 led to increased *Calx* expression and enhanced JNK amplification. Genetically, these promoted AiP through calcium signaling. Together, our findings revealed a microRNA-based regulatory mechanism that coordinates different stages of JNK activation during AiP.

**Supplementary Information:**

The online version contains supplementary material available at 10.1007/s00018-025-05880-w.

## Introduction

In multicellular organisms, stress-induced apoptosis can trigger cell proliferation, a phenomenon termed apoptosis-induced proliferation (AiP) [1, 2]. Initial studies in *Drosophila* have revealed that caspases, key proteases mediating apoptosis, also activate AiP via an induction of mitogenic growth signals such as Wg (*Drosophila* Wnt), Dpp (*Drosophila* TGF-β), Spi (*Drosophila* EGF), and Hedgehog (Hh) [3–8]. A similar function of caspases has been reported in *Hydra* and mouse to promote regeneration, wound healing, and tumorigenesis [9–12]. Studies in *Drosophila* have also revealed a central role of c-Jun N-terminal kinase (JNK) signaling, an evolutionarily conserved stress signaling, in AiP. It is activated downstream of caspases, leading to the induction of growth signals [3, 8, 13].

Intriguingly, recent studies have shown that JNK can be activated in apoptotic cells through several mechanisms. First, in response to apoptotic stress, an apical accumulation of actin filaments appears to be an early cellular process critical for AiP [14]. This actin cytoskeleton reorganization drives an elevation of cellular reactive oxygen species (ROS) which activates JNK cell-autonomously. Second, caspases can stimulate production of extracellular ROS via Duox, a NADPH oxidase [15–17]. These ROS disrupt the cellular basement membrane and recruit hemocytes, the *Drosophila* macrophages. Hemocytes produce Eiger, the *Drosophila* TNF (Tumor Necrosis Factor), to further promote JNK activation cell non-autonomously. Third, a signaling amplification loop also exists in apoptotic cells enhancing JNK activity [18]. In this loop, JNK signaling induces the expression of pro-apoptotic genes, hence further activation of caspases, JNK, and AiP. Little is known about how this process is regulated, although p53 and Toll signaling have been implicated in this process [19, 20].

MicroRNAs (miRNAs) are small (18 ~ 25 nucleotides) non-coding RNAs that regulate gene expression post-transcriptionally and play diverse roles in development [21]. Notably, miRNA genes often form clusters within the genome in both humans and flies, comprising members from the same or different families that are transcribed together [22–25]. It has been proposed that clustered miRNAs possess coordinated functions [26], however, this remains a topic of debate [27, 28]. Therefore, the in vivo functions of individual clustered miRNAs deserve further investigation. While miRNAs within the same cluster may regulate both shared and unique target genes, potentially influencing distinct cellular processes, there are limited reports of clustered miRNAs displaying distinct functions within the same cellular process.

The miR-309/3/286/4/5/6 − 1/6 − 2/6 − 3 (miR-309-6) cluster is the largest miRNA cluster in the *Drosophila* genome composed of eight members from four different miRNA families [29]. Although the evolutionary origin of this cluster remains unclear, miR-309/3/286/4/5 are believed to be more ancient, whereas miR-6-1/6 − 2/6 − 3 may arise from more recent duplications. Members of this cluster have been reported to share some common gene targets involved in *Drosophila* leg development [30]. However, whether individual miRNAs within this cluster exert different biological functions remains unknown.

In this study, we uncovered distinct functions of individual miRNAs within the miR-309-6 cluster, particularly miR-286 and miR-6, in regulating AiP. Loss of miR-6 or one copy of the entire miR-309-6 cluster inhibited the initial JNK activation during AiP. In contrast, overexpression of miR-286 suppressed the JNK-mediated induction of pro-apoptotic genes, which is essential for the feedback amplification of JNK signaling. Notably, we observed a decrease in miR-286 expression during AiP. Further analysis identified *Calx*, encoding a sodium/calcium (Na^+^/Ca^2+^) exchanger, as a direct target of miR-286 that promotes AiP. Consistent with this, CaMKII, a key kinase in the calcium signaling pathway, was found to mediate the roles of Calx and miR-286 in AiP, suggesting an involvement of calcium signaling in this process.

## Materials and methods

### Drosophila Genetics and stocks

All Genetic crosses were reared at 25°C unless otherwise specified. The *ey > hid-p35* stocks used for genetic screens and characterization of AiP-dependent overgrowth in adult heads and larval discs were as previously described [8, 14]. The full genotype of *ey > rpr-p35* is *ey-Gal4 UAS-p35/CyO; UAS-rpr/TM6 tub-Gal80*. The stocks *Df(2R)BSC22* (6647), *Df(2R)ED3728* (9067), *miR-309-6*^*∆1*^ (58922), *UAS-dronc*^*RNAi*^ (32963), *UAS-scramble-SP* (61501), *UAS-miR-309-SP* (61426), *UAS-miR-3-SP* (61369), *UAS-miR-4-SP* (61370), *UAS-miR-5-SP* (61371), *UAS-miR-6-SP* (61372), *UAS-miR-286-SP* (61418), *UAS-miR-6-1/2/3* (41136), *UAS-miR-286* (41151), *UAS-mCherry* (38424), *TRE-red* (59012), *Calx*^*A*^ (24496), *Calx*^*B*^ (24497), *UAS-Calx*^*RNAi*^ (28306), *UAS-CaMKII*^*RNAi*^ (35330), *UAS-CaMKII*^*T287D*^ (29664) and *UAS-lacZ* (8530) were obtained from the Bloomington *Drosophila* Stock Center. The *UAS-Calx* line was as previously described [31, 32].

### Mosaic analysis

Late 2nd instar larvae (64–72h post egg-laying) of the following Genotypes were heat shocked at 37°C for 7 min, then maintained at 25 °C for 48 h before dissection and analysis at the late 3rd instar stage. (1) Generation of clones co-expressing *hid* and *p35*: *hs-FLP/UAS-hid; act > y*^*+*^*>GAL4 UAS-GFP/+; UAS-p35/+*. (2) Generation of clones co-expressing *hid*, *p35* and *dronc*^*RNAi*^: *hs-FLP/UAS-hid; act > y*^*+*^*>GAL4 UAS-GFP/+; UAS-p35/UAS-dronc*^*RNAi*^. (3) Generation of clones co-expressing *hid*, *p35* in a *miR-309-6*^*∆1*^ mutant background: *hs-FLP/UAS-hid; act > y*^*+*^*>GAL4 UAS-GFP/miR-309-6*^*∆1*^; *UAS-p35/+*. (4) Generation of clones co-expressing *hid*, *p35* and *miR-6-SP*: *hs-FLP/UAS-hid; act > y*^*+*^*>GAL4 UAS-GFP/UAS-miR-6-SP; UAS-p35/UAS-miR-6-SP*. (5) Generation of clones expressing *hid*, *p35* and *miR-286*: *hs-FLP/UAS-hid; act > y*^*+*^*>GAL4 UAS-GFP/+; UAS-p35/UAS-miR-286*.

### Quantitative real-time PCR (qPCR) and TaqMan qPCR

Standard reverse transcriptase qPCR was used to measure *Calx* and *rpr* mRNA levels. Total RNA was extracted from 60 late 3rd instar larval eye discs (30 from females and 30 from males per genotype) using the QIAGEN RNeasy Plus Kit. cDNA was synthesized from 300ng of total RNA with the GoScript Reverse Transcription System (Promega). qPCR was performed using the SensiFAS SYBR Hi-ROX Kit (BIOLINE) on an Agilent AriaMx Real-Time PCR System. mRNA levels were normalized to the reference gene *ribosomal protein L32* (*RPL32*) by using the ΔΔCt analysis. Primers used for *Calx* [33], *rpr* [34], and *RPL32* [14] were as follows: *Calx* Fw, TTCGTGATCTTTGTGTATGCCA; *Calx* Rv, GAGGTAACATTAAGGTACGCCTG; *rpr* Fw, GAGCAGAAGGAGCAGCAGAT; *rpr* Rv, GGACTTTCTTCCGGTCTTCG; *RPL32* Fw, AGCATACAGGCCCAAGATCG; *RPL32* Rv, TGTTGTCGATACCCTTGGGC.

Expression levels of miR-6 and miR-286 were measured using TaqMan qPCR as described [30]. Total RNA was extracted from 400 late 3rd instar larval eye discs (200 from females and 200 from males per genotype) using the mirVana miRNA Isolation Kit (Invitrogen). cDNA was synthesized from 1µg total RNA using the TaqMan miRNA Reverse Transcription Kit (Applied Biosystems), followed by preamplification with the TaqMan PreAmp Master Mix Kit. qPCR was performed using TaqMan MicroRNA Assays (Applied Biosystems) on a QuantStudio 5 Real-Time PCR System. Statistical analyses of qPCR and TaqMan qPCR were performed using Student’s t-test, based on three independent biological repeats.

### Plasmid construction and luciferase reporter assay

The Actin5C-promoter DNA fragment from pAC5.1/V5-HisB vector (Life Technologies) was inserted into pGL3-basic plasmid (Promega) to generate Actin5C-firefly luciferase plasmid (pGL3-act5CLuc). The pAC5.1/V5-HisB-Renilla luciferase plasmid was constructed by cloning renilla luciferase from pRL-TK (Promega) into pAC5.1/V5-HisB vector. The *calx* 3’UTR fragments of 32 ~ 581nt, 1873 ~ 2401nt, and 3014 ~ 3499nt were PCR amplified, and cloned into XbaI site of pGL3-act5CLuc plasmid vector, respectively. Mutated *Calx* 3’UTR 3014 ~ 3499nt was generated by mutagenesis of the complementary *miR-286-3p* seed sequence from “GAUCU” to “AGCCG”. DNA fragments span the corresponding pre-miRNA locus, 233bp upstream and 172bp downstream of *pre-miR-286*, 172bp upstream and 215bp downstream of *pre-miR-309*, 373bp upstream and 107bp downstream of *pre-miR-6-1*, 214bp upstream and 155bp downstream of *pre-miR-6-2*, and 494bp upstream and 35bp downstream of *pre-miR-6-3*, were constructed into EcoRI/XhoI sites of pAC5.1/V5-HisB plasmid vector, respectively. Primers used for plasmid construction are listed in Supplemental Table S3.

The luciferase reporter assay was conducted as described [35]. *Drosophila* Schneider S2 cells were cultured in InsectPro Sf9 Medium with 5% heat-inactivated Fetal Bovine Serum (FBS) (Gibco) and 1% Pen-Strep Amphotericin B (Biological Industries) at 25°C. Plasmid DNA of 1µL *Calx* 3’UTR construct (100ng) or mutant *Calx* 3’ UTR construct (100ng), 1µL of pAC5.1-miRNA (300 ng) or pAC5.1-*lin-4* (300ng), and 1µL of pAC5.1-Renilla luciferase (50ng) for normalization were transfected using Effectene transfection reagent (QIAGEN). The luciferase activity was measured at 48 h post-transfection using the TransDetect Double-Luciferase Reporter Assay Kit (TransGen Biotech). Experiments were performed in triplicate, and statistical analysis was conducted using Student’s t-tests.

### Immunohistochemistry

Late 3rd instar larval eye and wing discs were dissected, fixed in 4% paraformaldehyde for 30 min at room temperature, followed by immunolabeling using standard protocols as described [36]. Primary antibodies used were rabbit anti-PH3 (1:1000, Merck 06-570), rat anti-ELAV (1:50, DHSB 7E8A10), mouse anti-MMP1 (all 1:50, DHSB 3A6B4, 3B8D12 and 5H7B1 used as a 1:1 cocktail) and rabbit anti-Hid (1:500, Santa Cruz sc-33744). Secondary antibodies were goat Fab fragments conjugated to Alexa 488, 555, or 647 (all 1:1000, Molecular Probes). Phalloidin (PHN, 1:40, Invitrogen R415) staining of the larval discs was performed at room temperature for one hour or co-incubated with secondary antibodies overnight at 4°C.

### Imaging, quantification, and statistical analysis

**Fig. 1:**
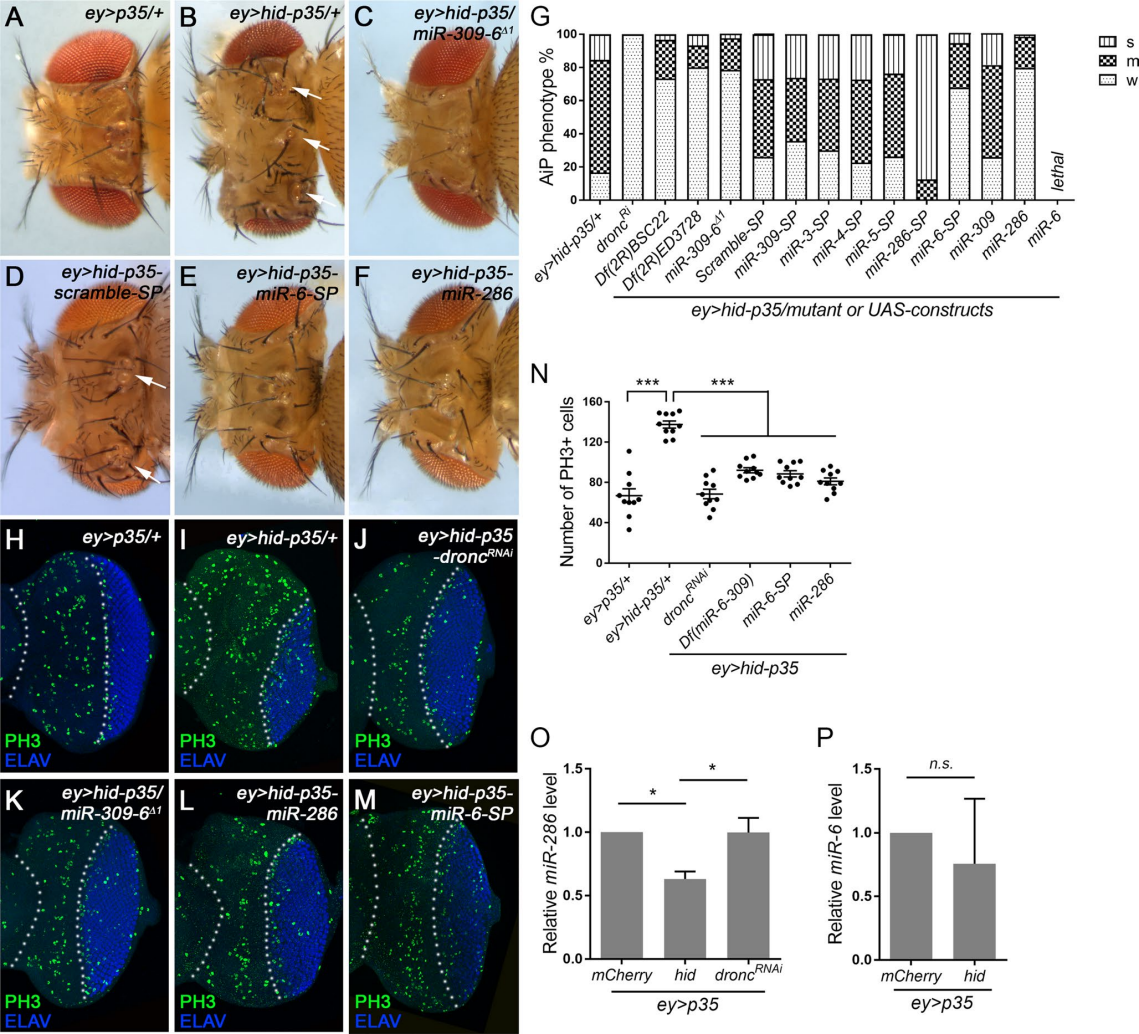
**miR-286 and miR-6 in the miR-309-6 cluster regulates AiP**. (A-F) Representative adult fly head images of the indicated genotypes. Compared to the control *ey > p35* (*ey-GAL4 UAS-p35*) (A), *ey > hid-p35* (*ey-GAL4 UAS-hid UAS-p35*) fly head capsules display overgrowth phenotypes which are grouped into three categories: severe (S), moderate (M) and weak (W, including wildtype-like), as previously described [8]. The majority of *ey > hid-p35* flies show moderate (68%) overgrowth phenotype, characterized by overgrown head capsules with duplications of sensory organs including bristles and ocelli (B, arrows). Loss of one copy of the miR-309-6 cluster (*miR-309-6*^*∆1*^) strongly suppresses the overgrown *ey > hid-p35* phenotype, as indicated by a large increase of flies (79%) showing a weak phenotype or wildtype-like appearance (C). Expression of the control *scramble-SP* has negligible effects on the *ey > hid-p35* overgrowth phenotypes (D, arrows). In contrast, expression of *miR-6-SP* (E) or miR-286 (F) strongly suppresses *ey > hid-p35* overgrown head phenotypes. (G) Summary of the effects on the *ey > hid-p35* overgrowth phenotype by the indicated genotypes. In each column, the top vertical line area, the middle dark grey region, and the bottom light dotted area indicate S, M, and W categories, respectively. (H-M) Late 3rd instar eye discs labelled with the mitotic marker PH3 (green) and the photoreceptor neuron marker ELAV (blue), anterior is to the left. ELAV is used to mark the posterior differentiating portion of the eye discs. The area between white dotted lines indicates the anterior proliferating portion of the eye discs. Compared to the control *ey > p35* (H), the size of the anterior proliferating portion and the number of mitotic cells increase in the *ey > hid-p35* eye discs (I). These increases are suppressed by expressing *dronc*^*RNAi*^ (J), *miR-309-6*^*∆1*^ heterozygous mutants (K), expressing miR-286 (L) or *miR-6-SP* (M). (N) Quantification of the number of PH3^+^ cells in the anterior portion of the eye discs (*n* = 10 per genotype). One-way ANOVA with Bonferronis’s multiple comparison test was used to compute p-values. Compared to *ey > p35*, the number of PH3^+^ cells are significantly increased in the *ey > hid-p35* discs (***p < 0.001). This increase is suppressed by *dronc*^*RNAi*^, *miR-309-6*^*∆1*^, miR-286, or *miR-6-SP* (***p < 0.001). (O, P) Expression levels of miR-286 (O) and miR-6 (P). Unpaired Student’s t-tests were used to compute p-values. Compared to the control, miR-286 is significantly reduced in *ey > hid-p35*, which is restored by *dronc*^*RNAi*^ (O, *p < 0.05). In contrast, miR-6 does not show significant changes in *ey > hid-p35* (P, n.s.=not significant)

**Fig. 2:**
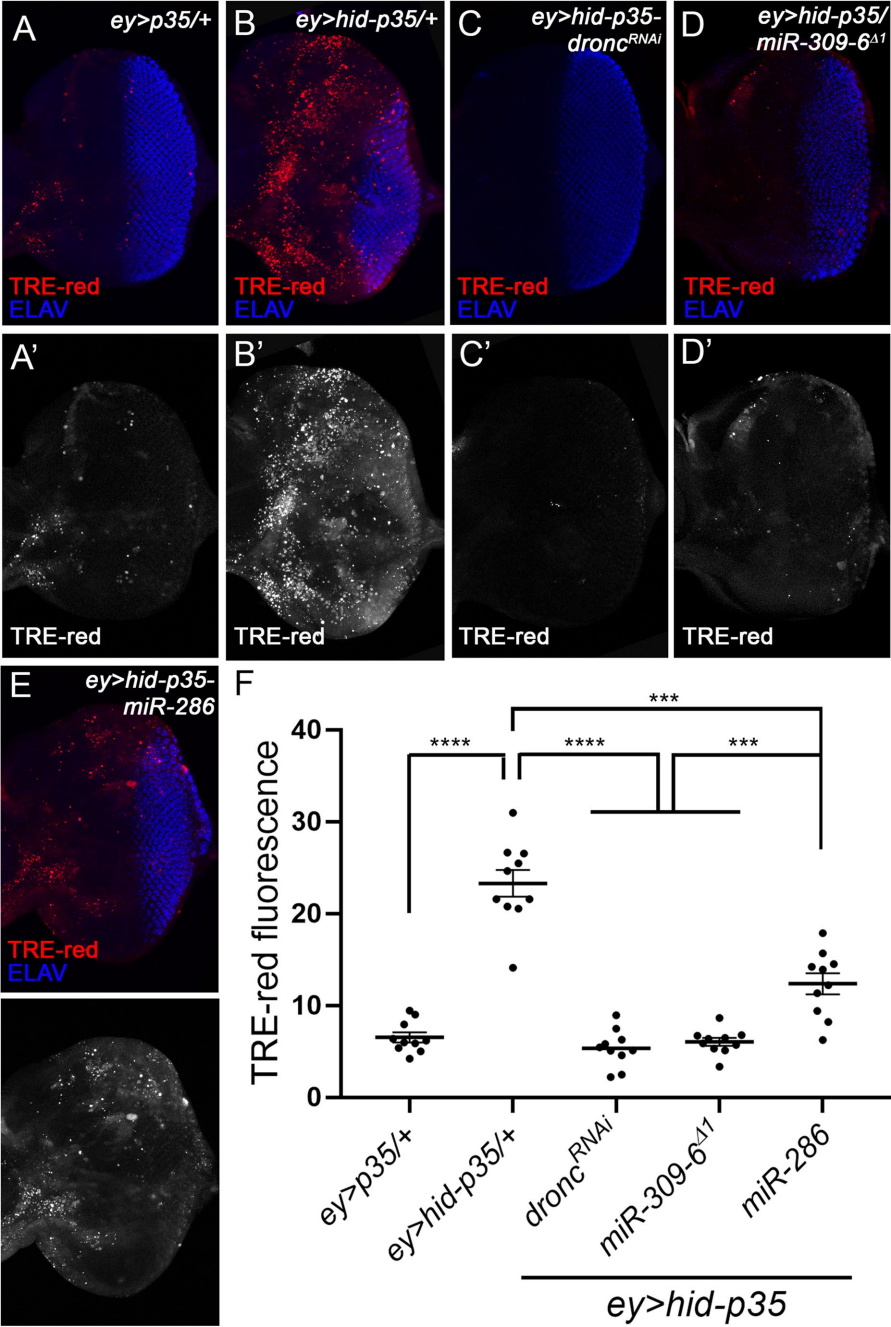
**Loss one copy of the miR-309-6 cluster or overexpression of miR-286 inhibits JNK activation in** ***ey > hid-p35*** **discs.** (A-E’) Late 3rd instar eye discs labelled with *TRE-red* (red in A-E and grey in A’-E’), a marker of JNK activity, and ELAV (blue in A-E). Compared to the control *ey > p35* (A, A’), *TRE-red* signals strongly increase in the *ey > hid-p35* eye discs (B, B’). This increase is suppressed by expressing *dronc*^*RNAi*^ (C, C’), heterozygous *miR-309-6*^*∆1*^ (D, D’), and, to a less extend, expression of miR-286 (E, E’). (F) Quantification of the *TRE-red* signal intensity in the eye discs (*n* = 10 per genotype). One-way ANOVA with Bonferronis’s multiple comparison test was used to compute p-values. Compared to the control *ey > p35*, the *TRE-red* signals are significantly increased in the *ey > hid-p35* discs (****p < 0.0001). This increase is strongly reduced (****p < 0.0001) in response to expression of *dronc*^*RNAi*^ or loss of one copy of the miR-309-6 cluster, but to a less extent (***p < 0.001) in response to overexpression of miR-286

**Fig. 3:**
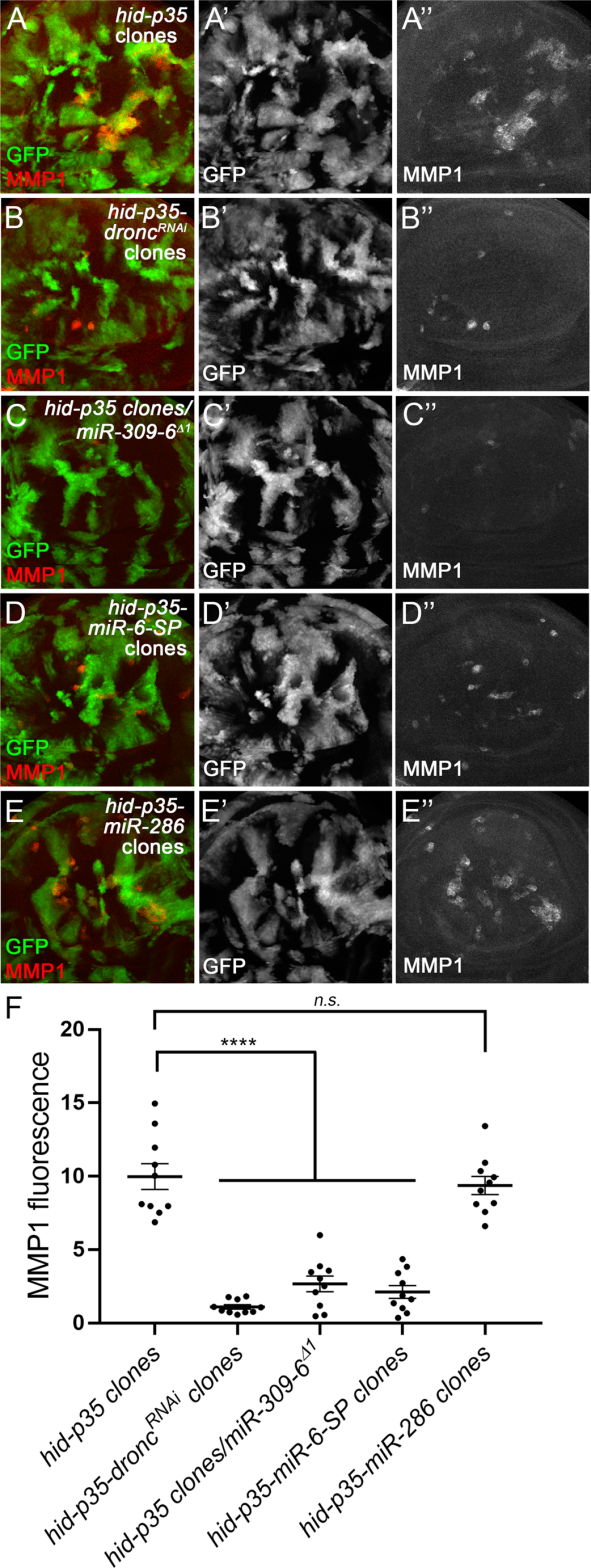
**Loss of miR-6**,** but not overexpression of miR-286**,** suppresses JNK activation in** ***hid*** **and** ***p35*** **expressing clones.** (A-E’’) Late 3rd instar wing discs with 48-hour-old mosaic clones positively marked by GFP (green in A-F and grey in A’-F’). MMP1 (red in A-F and grey in A’’-F’’) is a marker of JNK activity. A strong level of MMP1 labelling was observed in the clones simultaneously expressing *hid* and *p35* (*hid-p35* clones) (A-A’’). It is largely inhibited by expression of *dronc*^*RNAi*^ (B-B’’), loss one copy of the miR-309-6 cluster (C-C’’), or expression of *miR-6-SP* (D-D’’). In contrast, overexpression of miR-286 has no effects on the *hid-p35*-induced MMP1 expression (E-E’’). (F) Quantification of the MMP1 signal intensity in clones in the representative wing discs (n = 10 per genotype). One-way ANOVA with Bonferronis’s multiple comparison test was used to compute p-values. Compared to the discs with *hid-p35* clones, the MMP1 signals are significantly (****p < 0.0001) suppressed in response to expression of *dronc*^*RNAi*^, loss one copy of the miR-309-6 cluster, or expression of *miR-6-SP*, but not expression of miR-286

**Fig. 4:**
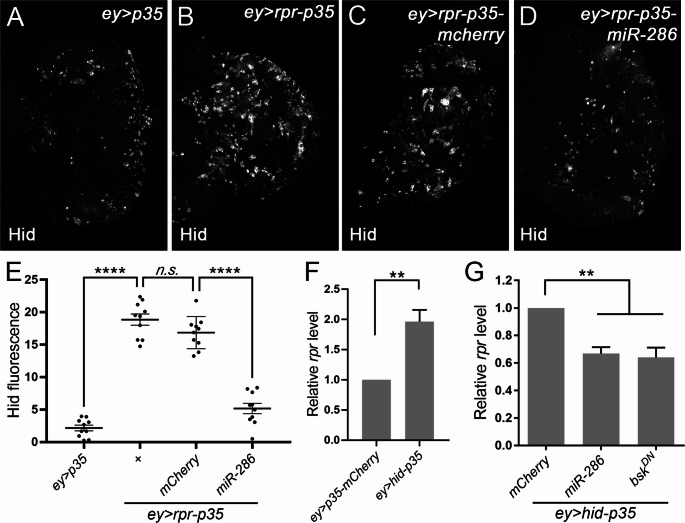
**Overexpression of miR-286 inhibits JNK-dependent transcriptional regulation of pro-apoptotic genes**. (A-D) Late 3rd instar eye discs labelled with Hid antibodies. Compared to the control *ey > p35* (A), expression of Hid is strongly induced in *ey > rpr-p35* (**B**) and *ey > rpr-p35-mCherry* (**C**) discs. This increase is suppressed by expression of miR-286 (**D**). (**E**) Quantification of Hid expression in the corresponding genotypes (****p < 0.0001). One-way ANOVA with Bonferronis’s multiple comparison test was used to compute p-values. (F, G) Expression levels of *rpr* measured by RT-qPCR. Unpaired Student’s t-tests were used to compute p-values. Compared to the control, expression of *rpr* is increased about 2 folds in *ey > rpr-p35* (F, **p < 0.01). This increase is suppressed to a similar level in response to expression of miR-286 or *bsk*^*DN*^, a dominant negative mutant of JNK (G, **p < 0.01)

Adult fly head images were acquired using a Zeiss Stemi 2000-CS stereomicroscope equipped with an AxioCam ICC1 camera. Fluorescent images of larval eye and wing discs were taken with a Zeiss LSM 710 confocal microscope. The number of PH3-positive cells (Fig. 1N) and the fluorescence intensity of TRE-red signals (Fig. 2F) and Hid staining (Fig. 4E) were quantified using representative larval eye discs (*n* = 10 for each genotype) as described [14]. Statistical analyses for cell counting and fluorescent signal intensity were conducted with GraphPad Prism 7 using a one-way ANOVA with Bonferronis’s multiple comparison test and plotted with Mean ± SEM. For quantification of PH3-positive cells (Fig. 1N), representative late 3rd instar larval eye discs (*n* = 10 per genotype) were scored using ImageJ with the Cell Counter plugin. Quantification of fluorescence intensity for TRE-red signals (Fig. 1F) and Hid staining (Fig. 4E) in larval eye discs and MMP1 labeling in mosaic analysis (Fig. 3F) was performed using ImageJ. For each sample (*n* = 10 per genotype), fluorescence intensity was measured in a defined region of interest (ROI) and a corresponding background area. The normalized intensity was calculated by subtracting the mean background signal from the ROI signal. ROIs and background areas were selected using ImageJ drawing tools as follows: (1) For TRE-red and Hid staining, the ROI was the entire eye disc; background was measured in the adjacent antenna disc. (2) For MMP1 signals, the ROI was the whole wing pouch; background was taken from non-clonal regions within the same pouch.

## Results

### miR-286 and miR-6 in the miR-309-6 cluster possess opposite roles in regulation of AiP

We previously developed a *Drosophila* assay that enables convenient screening of AiP regulators [8]. In this assay, the pro-apoptotic gene *hid* and the caspase inhibitor *p35* are co-expressed under the control of the eye-specific driver *ey-GAL4*, i.e., the *ey > hid-p35* assay. Since P35 inhibits Caspase-3-like caspases DrICE and Dcp-1, it effectively blocks apoptosis, but not AiP, which relies on Dronc, the caspase-9-like caspase acting upstream of DrICE and Dcp-1. As a result, the *ey > hid-p35* assay induces overgrowth in developing eye tissue, resulting in adult fly heads with expanded head cuticles and ectopic sensory organs such as ocelli and bristles (Fig. 1B, compared to 1A). Using this assay, we performed a genetic modifier screen with chromosomal deficiencies deleting various genomic regions to search for potential regulators of AiP [8]. We found that heterozygosity of the deficiency *Df(2R)BSC22* or *Df(2R)ED3728*, both remove the miR-309-6 cluster, suppressed the *ey > hid-p35* head overgrowth phenotype (Fig. 1G). Similarly, *miR-309-6*^*∆1*^, a small deletion specifically removing the miR-309-6 cluster [37], also exhibited a suppressive effect (Fig. 1 C and G). These results suggest that the miR-309-6 cluster is required for AiP.

To determine which miRNA(s) within the miR-309-6 cluster regulates AiP, we performed a loss-of-function analysis using miRNA-sponge (miRNA-SP) constructs designed to competitively bind and sequester individual miRNAs, thereby inhibiting their activities [38]. Expression of *scramble-SP*, a control SP, did not affect the *ey > hid-p35*-induced head overgrowth phenotype (Fig. 1D and G). Similarly, expressions of *miR-309-SP*, *miR-3-SP*, *miR-4-SP*, or *miR-5-SP* yielded results comparable to the control (Fig. 1G). In contrast, *miR-286-SP* and *miR-6-SP* significantly impacted the *ey > hid-p35* phenotype: *miR-286-SP* enhanced overgrowth, whereas *miR-6-SP* suppressed it (Fig. 1E and G). Consistent with these findings, overexpression of miR-286 strongly suppressed the *ey > hid-p35* phenotype (Fig. 1F and G), while overexpression of miR-6 in *ey > hid-p35* led to lethality, suggesting a strong enhancing effect on AiP (Fig. 1G). Notably, expression of either *miR-286-SP* or miR-6 alone by *ey-GAL4* did not result in any observable developmental defects (Supplemental Figure S1). Thus, the effects of *miR-286-SP* or miR-6 on the *ey > hid-p35* phenotype likely reflect their specific roles in promoting AiP.

In addition to adult fly heads, we examined developing larval eye tissues. Compared to the control *ey > p35* (Fig. 1H and N), an increase of cell proliferation was observed in overgrown *ey > hid-p35* larval eye discs (Fig. 1I and N). This increase as well as tissue overgrowth was suppressed by RNAi knockdown of Dronc, the initiator caspase required for AiP (Fig. 1J and N). Consistent with adult phenotypes, both the increased proliferation and overgrowth in *ey > hid-p35* larval eye tissues were suppressed by loss of one copy of the miR-309-6 cluster, as well as by expression of miR-286 or *miR-6-SP* (Fig. 1K and N). Similar suppression of AiP-dependent tissue overgrowth was also observed in developing larval wing discs (Supplemental Figure S2). These results indicate that miR-6 is required for AiP, whereas miR-286 inhibits it. Despite their opposing roles, loss of the entire cluster, as seen in heterozygous *miR-309-6*^*∆1*^ mutants, suppressed AiP, phenocopying the effect of *miR-6-SP* (Fig. 1G and N). While this does not rule out contributions from other cluster members, the similarity between miR-6 loss and cluster deletion suggests that miR-6 plays a dominant role in promoting AiP. We therefore focused our study on the distinct and opposing roles of miR-6 and miR-286 in AiP.

To further investigate how these miRNAs are involved in AiP, we examined whether the levels of mature miR-286 and miR-6 are regulated during this process. In *ey > hid-p35* larval eye tissues, a notable decrease in miR-286 abundance was observed compared to the control (Fig. 1O). This reduction was blocked by RNAi knockdown of Dronc, the key coordinator of apoptosis and AiP. In contrast, miR-6 levels did not show significant change, although considerable variability was detected, possibly due to its relatively low expression level (Fig. 1P). Hence, expression of miR-286, but possibly not miR-6, is regulated during AiP.

### miR-6, but not miR-286, regulates the initial activation of JNK in AiP

Loss of miR-6 has been shown to regulate apoptosis during embryonic development [39, 40], potentially explaining its involvement in AiP. However, neither heterozygous *miR-309-6*^*∆1*^, expression of *miR-6-SP*, nor miR-286 overexpression affected *hid*-induced apoptosis in larval eye discs (Supplemental Figure S3). To investigate how miR-286 and miR-6 function in AiP, we examined whether they regulate the activation of JNK, a key signaling event that triggers AiP. Using a JNK reporter TRE-red [41], we observed strong JNK activation in *ey > hid-p35* larval eye discs (Fig. 2B, compared to 2A). As expected, this activation was suppressed by RNAi knockdown of Dronc (Fig. 2C). Although we could not analyze the effect of *miR-6-SP* expression on JNK activation in this assay because the SP constructs carry mCherry, which interferes with TRE-red detection, we found that loss of one copy of the miR-309-6 cluster significantly suppressed both JNK activation and tissue overgrowth in *ey > hid-p35* discs (Fig. 2D and F). Interestingly, overexpression of miR-286 also suppressed tissue overgrowth. However, its inhibition of JNK was relatively modest and significantly weaker than the suppression caused by *dronc*^*RNAi*^ or heterozygous *miR-309-6*^*∆1*^ (Fig. 2E and F). Since overexpression of miR-286 effectively blocks tissue overgrowth, the reduced JNK activity may reflect the reduction of tissue overgrowth itself, rather than direct JNK regulation by miR-286.

To further examine how miR-286 impacts JNK during AiP, we conducted clonal analysis, which allows observation of the initial JNK activation that triggers AiP without the confounding effect of tissue overgrowth [14]. In this approach, *hid* and *p35* were co-expressed in clones for 48 h, resulting in strong JNK activation within the clones but no tissue overgrowth (Fig. 3A). RNAi knockdown of Dronc, expression of *miR-6-SP* or heterozygous loss of the miR-309-6 cluster all suppressed JNK activation in these clones (Fig. 3B-D). In contrast, expression of miR-286 did not noticeably affect JNK activation (Fig. 3E and F). Together, these data suggest that miR-6, but not miR-286, is required for the initial activation of JNK that triggers AiP. In contrast, miR-286 may act at a later stage to restrain amplification of JNK signaling, a process critical for driving tissue overgrowth when apoptosis is inhibited.

### miR-286 regulates the feedback amplification of JNK in AiP

It has been reported that JNK, once activated in response to apoptotic stress, can further amplify its activity through a feedback loop that enhances the expression of pro-apoptotic genes, including *hid* and *rpr* [18]. To investigate whether miR-286 regulates this process, we measured Hid protein levels in eye discs where *rpr* and *p35* were co-expressed under *ey-GAL4* control (*ey > rpr-p35*). As expected, compared to *ey > p35* (Fig. 4A), Hid levels were strongly increased in *ey > rpr-p35* discs (Fig. 4B and E). Expression of a control *mCherry* construct had no noticeable effect on Hid levels in this context (Fig. 4C and E). In contrast, miR-286 expression significantly suppressed the elevated Hid (Fig. 4D and E). Similarly, *rpr* expression was induced in *ey > hid-p35* discs (Fig. 4F), and this induction was suppressed by miR-286, to a degree comparable to *bsk*^*DN*^, a dominant-negative mutant form of the *Drosophila* JNK (Fig. 4G). These findings suggest that miR-286 regulates JNK-driven *hid* and *rpr* expression in response to stress.

### Calx, a Na^+^/Ca^2+^ exchanger, is identified as a miR-286 target required for AiP

Since miR-286 levels are regulated during AiP, we sought to identify its target genes that are functionally important for JNK-dependent expression of *hid* and *rpr*, as well as for AiP. To achieve this, we used three online computational tools to predict miR-286 targets in *Drosophila*: TargetScanFly (https://www.targetscan.org/fly_72/) [42–44], PicTar (https://pictar.mdc-berlin.de/) [45, 46], and miRanda (microRNA.org, http://mirtoolsgallery.tech/mirtoolsgallery/node/1055) [47–49]. These tools predicted 982, 120, and 391 target genes, respectively (Supplemental Table S1). Among them, TargetScan is unique in distinguishing between 5p and 3p miRNAs, predicting 764 targets for miR-286-5p and 218 for miR-286-3p. The predicted results from these three tools varied considerably, likely due to differences in their underlying algorithms, suggesting a substantial number of false positives. To refine our analysis, we identified the overlapping targets predicted by these tools. Notably, although TargetScan predicted more targets for miR-286-5p than for miR-286-3p, only 12 overlapped with PicTar and miRanda for miR-286-5p, compared to 71 for miR-286-3p (Supplemental Table S2). Interestingly, 10 of these 12 were also found within the 71.

**Fig. 5:**
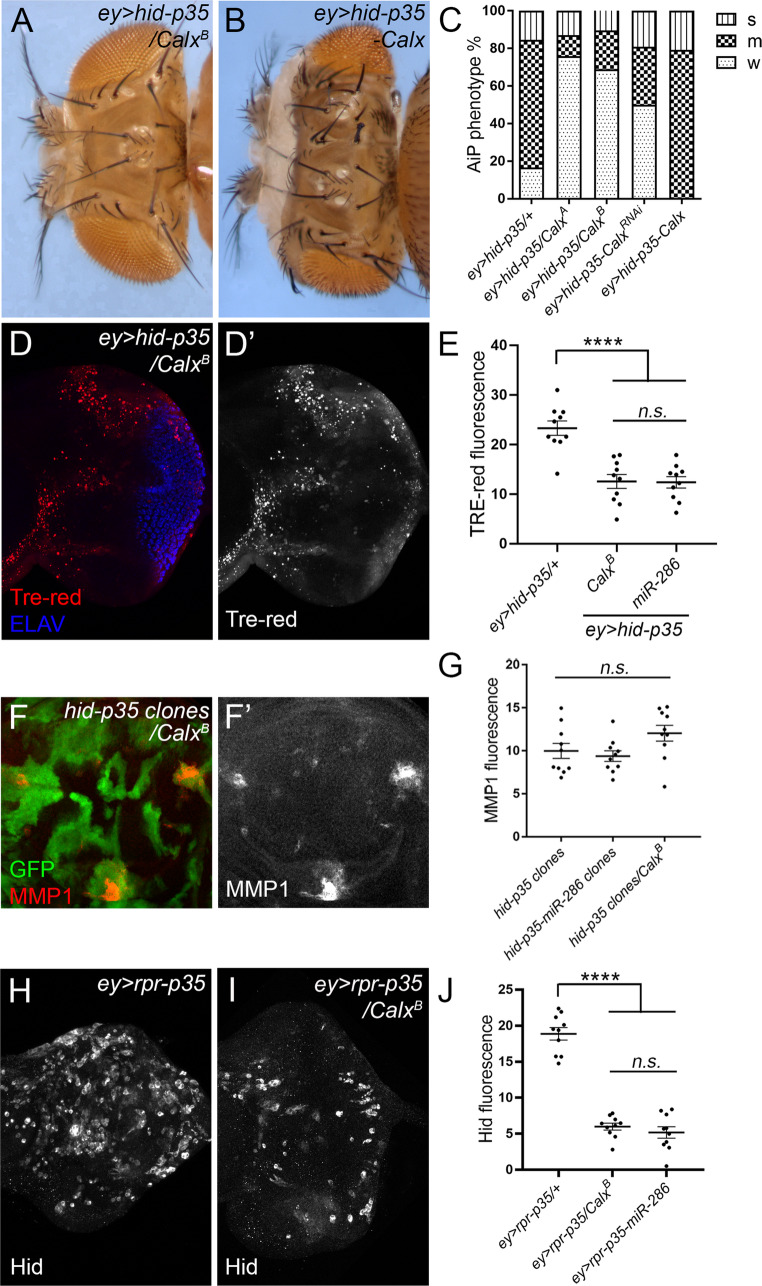
**Calx is required for AiP through its regulation of JNK amplification but not initiation**. (A-C) Representative adult fly head images and genetic screen results showing heterozygous *Calx*^*B*^ mutants suppress the *ey > hid-p35* overgrowth phenotype (A, compared to Fig. 1B) and overexpression of *Calx* enhances it (**B**), as indicated by summary of the phenotypic categories of the indicated genotypes (**C**). (**D,** **E**) *ey > hid-p35*-induced TRE-red signals are partially suppressed in heterozygous *Calx*^*B*^ mutants (D, D’) and quantified in (**E**) showing a similar effect compared to expression of miR-286 (****p < 0.0001, n.s.=not significant). (**F, G**) Elevated MMP1 signals in *hid-p35* clones are not suppressed by heterozygous *Calx*^*B*^ mutants (F, F’) and quantified in (**G**) showing a similar effect compared to expression of miR-286. (H-J) Elevated Hid expression in *ey > rpr-p35* (**H**) is suppressed by expression of the heterozygous *Calx*^*B*^ mutants (**I**) and quantified in (**J**) showing a similar effect compared to expression of miR-286. One-way ANOVA with Bonferronis’s multiple comparison tests were used to compute p-values

Among these 10 overlapping targets, *Calx*, which encodes a Na^+^/Ca^2+^ exchanger [50–52], was identified as a key regulator of AiP using the *ey > hid-p35* assay. Heterozygous loss-of-function mutants *Calx*^*A*^ or *Calx*^*B*^ [31], as well as RNAi knockdown of *Calx*, suppressed the *ey > hid-p35*-induced overgrowth phenotype (compare Fig. 5A to Fig. 1B, quantified in Fig. 5C). In contrast, *Calx* overexpression enhanced this phenotype, as indicated by the increased percentage of flies showing overgrown heads (Fig. 5B and C). These findings suggest that *Calx* is required for AiP, and that its overexpression further promotes the process. Consistent with *Calx* being a potential target of miR-286, loss of *Calx*, using the *Calx*^*B*^ mutant which exhibits a severe reduction in Calx protein levels [53], partially inhibited JNK activation in *ey > hid-p35* whole discs (Fig. 5D and D’ and E). Similar to overexpression of miR-286, *Calx* loss had no obvious effect on JNK activation induced by *hid-p35*-expressing clones (Fig. 5F and F’ and G). Moreover, loss of *Calx* suppressed *rpr*-induced *hid* expression, mimicking the consequence of miR-286 overexpression (Fig. 5H and I and J).

**Fig. 6:**
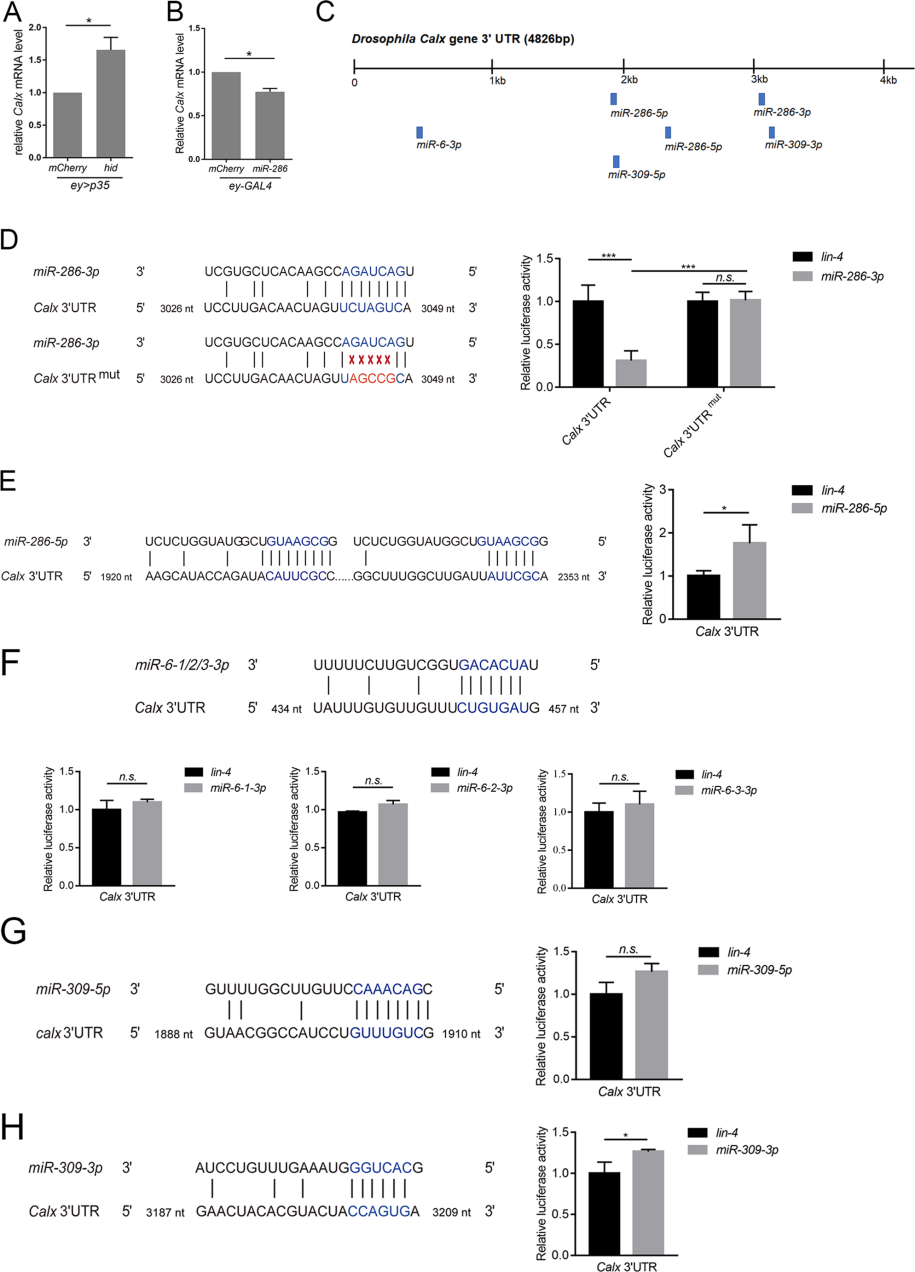
**Calx is a direct target of miR-286**. (A, B) Expression levels of *Calx* are measured by RT-qPCR for the indicated genotypes. Compared to the control, *Calx* expression is increased by about 1.7 fold in *ey > hid-p35* (A, *p* < 0.05). Expression of *Calx* is reduced by about 23% in response to miR-286 expression (B, *p* < 0.05). (**C**) A diagram indicating the distribution of potential targeting sites (blue bars) of individual miRNAs in about 5 kb of the *Calx* 3’ UTR. (D-H) Seed sequences of the indicated miRNAs in the 3’UTR of *Calx* and measurement of corresponding luciferase assays. Expression of miR-286-3p significantly (**p < 0.01) suppresses *Calx* 3’UTR-mediated luciferase activity, which is abolished by the mutant seed sequence (**D**). In contrast, expression of miR-286-5p (**E**), miR-6-1/2/3-3p (**F**), miR-309-5p (**G**) and miR-309-3p (**H**) does not reduce *Calx* 3’UTR-mediated luciferase activity. Unpaired Student’s t-tests were used to compute p-values

As microRNAs often regulate gene expression by promoting target mRNA degradation, we measured *Calx* mRNA levels in *ey > hid-p35* whole discs and assessed its response to miR-286 overexpression. Compared to the *ey > p35-mCherry* control, *Calx* expression was elevated in *ey > hid-p35* discs (Fig. 6A), while miR-286 overexpression reduced *Calx* levels (Fig. 6B), suggesting that miR-286 promotes *Calx* mRNA degradation. To determine whether *Calx* is a direct target of miR-286, we searched the 5 kb 3’UTR of *Calx* using TargetScanFly and identified potential binding sites for miR-286, miR-6, and miR-309 of the miR-309-6 cluster, including two predicted sites for miR-286-5p and one for miR-286-3p (Fig. 6C). We then performed a dual-luciferase reporter assay in *Drosophila* S2 cells to test the responsiveness of these sites to their corresponding miRNAs. Expression of miR-286-3p, but not other miRNAs including miR-286-5p, significantly reduced the luciferase activity of a reporter containing the wild-type *Calx* 3′UTR (Fig. 6D - H). The repression was abolished when a reporter containing a mutated *Calx* 3′UTR was co-transfected with the miR-286-3p construct (Fig. 6D). These results suggest that *Calx* is a putative target of miR-286-3p. Together, our data identify miR-286 as a regulator of AiP through its targeting of *Calx*.

### Calcium signaling mediates the roles of miR-286 and Calx in AiP

**Fig. 7:**
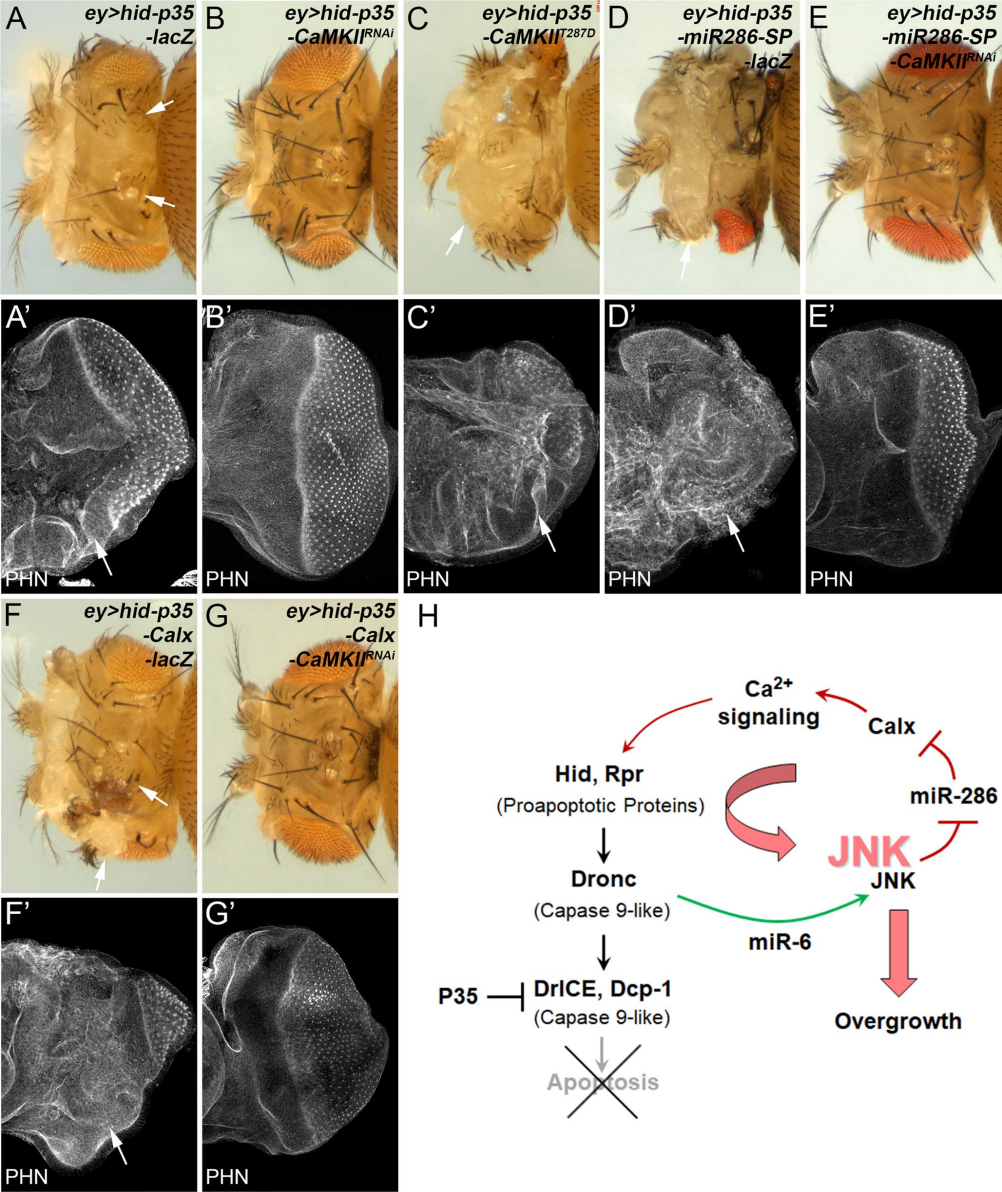
**miR-286 and Calx promote AiP through calcium signaling**. (A-G’) Representative adult head images of the indicated genotypes (A-G) and their corresponding late 3rd instar eye discs (A’-G’) labelled with Phalloidin (PHN), a marker of F-actin indicating disc morphology. Compared to the control *ey > hid-p35-lacZ* (A, A’), the overgrowth in adult heads as well as eye discs is strongly suppressed by RNAi knockdown of CaMKII (B, B’) but is enhanced by expression of a constitutively active mutant of *CaMKII*, *CaMKII*^*T287D*^ (C, C’). Enhancing effects of miR-286 on *ey > hid-p35* phenotypes (D, D’) are inhibited by knockdown of CaMKII (E, E’). Similarly, enhancing effects of Calx on *ey > hid-p35* phenotypes (F, F’) are inhibited by knockdown of CaMKII (G, G’). (H) A diagram summarizing our findings of miR-6 and miR-286 in regulation of JNK. While miR-6 is required for the initial activation of JNK (indicated in green), miR-286 regulates JNK amplification (indicated in red) through Calx and calcium signaling, thereby promoting tissue overgrowth

The Na^+^/Ca^2+^ exchanger Calx plays a critical role in maintaining intracellular calcium homeostasis in various contexts, including phototransduction and circadian rhythms [31, 54, 55]. We therefore investigated whether the role of Calx in AiP is mediated by calcium signaling. Indeed, RNAi knockdown of Ca^2+^/calmodulin-dependent protein kinase II (CaMKII), a key factor that couples intracellular calcium signals to diverse cellular responses [56, 57], suppressed the *ey > hid-p35* overgrowth phenotype (Fig. 7A and B). Consistently, expression of *CaMKII*^*T287D*^, a constitutively active mutant of *CaMKII* [58, 59], enhanced the phenotype (Fig. 7C), supporting a role for calcium signaling in driving AiP. Notably, the *miR-286-SP*-enhanced *ey > hid-p35* overgrowth phenotype was also suppressed by RNAi knockdown of CaMKII (Fig. 7D and E), suggesting that miR-286 acts upstream of calcium signaling in this context. Consistent with the notion that *Calx* is a target of miR-286, *Calx* overexpression enhanced the *ey > hid-p35* phenotype (Fig. 7F), and this enhancement was inhibited by *CaMKII* RNAi (Fig. 7G). Taken together, these findings support a model in which miR-286 regulates AiP through Calx-dependent modulation of calcium signaling (Fig. 7H).

## Discussion

In this study, we identified distinct roles for miR-286 and miR-6, two members of the miR-309-6 cluster, in regulating AiP. While miR-6 mediates the initial activation of JNK in response to apoptotic stress, miR-286 is specifically required for the further amplification of JNK activity. This occurs through the regulation of pro-apoptotic genes via its target, *Calx*, and modulation of calcium signaling (Fig. 7H). These findings underscore the critical role of JNK in AiP and highlight the multiple layers of control involved in this process. It has been proposed that when the execution of apoptosis is blocked, such as by expression of the effector caspase inhibitor P35, the initiator caspase Dronc continuously drives AiP through JNK activation and amplification, ultimately leading to tissue overgrowth [14, 15]. Our observation of the distinct roles of miR-286 and miR-6 further supports this model.

An important question is how these two clustered miRNAs execute different functions. miR-286 and miR-6 belong to different miRNA families and possess distinct seed sequences, enabling them to regulate different target genes. Our findings suggest that miRNAs within the same cluster can coordinate their activities to regulate distinct aspects of a shared biological process. We identified *Calx* as a direct target of miR-286. As a Na^+^/Ca^2+^ exchanger, Calx is a critical regulator of intracellular calcium signaling [31, 54, 55]. Our genetic analysis suggests that Calx mediates AiP through calcium-dependent transcriptional regulation of the pro-apoptotic genes *hid* and *rpr*. Notably, calcium signaling can influence gene transcription by regulating various stages directly, including initiation, elongation and termination, or indirectly via epigenetic mechanisms [60, 61]. In *Drosophila*, *hid* and *rpr* expression are subject to transcriptional and epigenetic regulation in response to stressors such as ionizing radiation [62–64]. It will be interesting to explore whether calcium signaling regulates these processes in the context of AiP. Additionally, both *p53* and Toll signaling have been implicated in JNK-dependent regulation of *hid* and *rpr* [18–20]. How miR-286 interfaces with these pathways remains an open question.

Unlike miR-286, miR-6 is required for the initial activation of JNK in stressed cells. In this study, we have not yet identified direct targets of miR-6 involved in AiP. Interestingly, miR-6 is known to repress apoptotic cell death during *Drosophila* embryonic development [39]. Loss of miR-6 leads to increased expression of pro-apoptotic genes such as *hid*, *rpr*, *grim* and *sickle*, all of which contain predicted miR-6 binding sites within their 3’UTRs [40]. However, this function does not explain the role of miR-6 in AiP observed in larval epithelial tissues, where *miR-6-SP* expression does not affect apoptosis. This suggests that miR-6 has context-dependent functions. Identifying miR-6 target gene(s) that function specifically in AiP will provide valuable mechanistic insights. Given that actin remodeling is a key step in initiating JNK activation during AiP, it is worth investigating whether miR-6 modulates the actin cytoskeleton in this context. miRNAs are known to regulate actin cytoskeleton dynamics by affecting expression of actin genes directly or of genes critical for actin remodeling [65]. Notably, among ~ 900 genes predicted as potential miR-6 targets using TargetScan, over 100 encode actin-binding proteins [30]. However, due to the high false-positive rate in miRNA target prediction, further functional analysis is needed to confirm their in vivo relevance in AiP.

Another notable finding is that the expression of miR-286, but not miR-6, is regulated during AiP. The miR-309-6 cluster has been reported to be transcribed as a single polycistronic transcript. However, differential expression of individual cluster members has been observed across various developmental stages, including larval stage, based on analyses of ModENCODE small RNA datasets [30]. This suggests that post-transcriptional mechanisms regulate the expression levels of these miRNAs, potentially through differential processing of pre-miRNAs or variation in the stability of mature miRNAs [66, 67]. Nevertheless, it remains unclear whether and how individual miRNAs within the miR-309-6 cluster are differentially processed or degraded. Our findings suggest that the differential regulation of miR-286 and miR-6 maturation or stability is critical for AiP. Importantly, this regulation appears to be context-dependent. For example, members of the miR-309-6 cluster show similar functions in promoting leg epithelial development, despite their expression levels vary [30]. How clustered miRNAs evolve and coordinate their functions remains an intriguing question. It has been suggested that miR-6 arose from a more recent duplication within the miR-309-6 cluster [29, 68, 69]. Notably, loss of miR-6 phenocopies the loss of the entire cluster in promoting AiP, while miR-286, despite being in the same cluster, can be differentially regulated to suppress AiP. This suggests a possible self-regulatory mechanism within miRNA clusters, allowing them to embed internal checks and balances to prevent detrimental effects. Such an evolutionary strategy for embedding self-constraint mechanisms within miRNA clusters warrants further investigation.

In conclusion, we have revealed the critical but distinct roles of clustered miR-6 and miR-286 in JNK activation and amplification during AiP. We also identified *Calx* as a direct target of miR-286, mediating AiP through its role in regulating calcium signaling. In future work, it will be interesting to identify the specific targets of miR-6 involved in AiP, investigate how the expression of miR-286, miR-6, and other cluster members is differentially regulated, and explore how this regulation impacts their physiological roles.

## Supplementary Information

Below is the link to the electronic supplementary material.

Supplementary Material 1 (DOCX 803 KB)

## Data Availability

Data sharing is not applicable to this article as no datasets were generated or analyzed during the current study. The manuscript has data included as electronic supplementary material.
