# Supplementary material for: Distinct roles of clustered MicroRNAs miR-286 and miR-6 in JNK activation critical to apoptosis-induced proliferation in Drosophila
Source: Cell Mol Life Sci. 2025 Nov 25;82(1):415. doi: 10.1007/s00018-025-05880-w (PMC12647430; doi:10.1007/s00018-025-05880-w)
Supplement: Supplementary file 1 — Supplementary Material 1 (DOCX 803 KB) [file 18_2025_5880_MOESM1_ESM.docx]

**Distinct roles of clustered microRNAs miR-286 and miR-6 in JNK activation critical to apoptosis-induced proliferation in *Drosophila***

*Mengyuan Yu, Caitlin Hounsell, Buyun Zhang, Tingxuan Wang, Xiaolin Bi, Yun Fan*

**Supplementary Information (Figures S1-S3 and Tables S1-S3)**

**
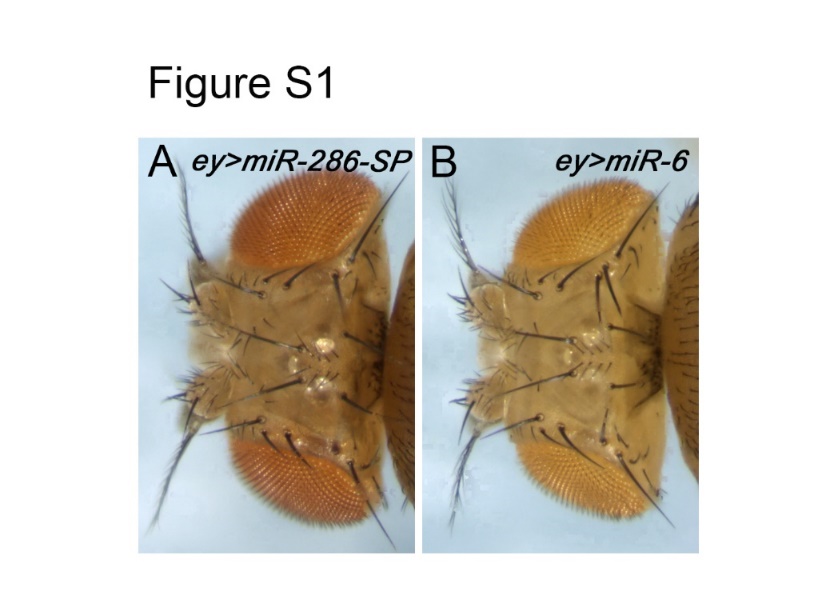
**

**Supplemental Figure S1. Overexpression of miR-286-SP or miR-6 does not lead to any developmental defects.**

(A, B) Representative adult head images of the indicated genotypes. Expression of miR-286-SP (A) or miR-6 (B) under the control of *ey-GAL4* does not cause any phenotypes.


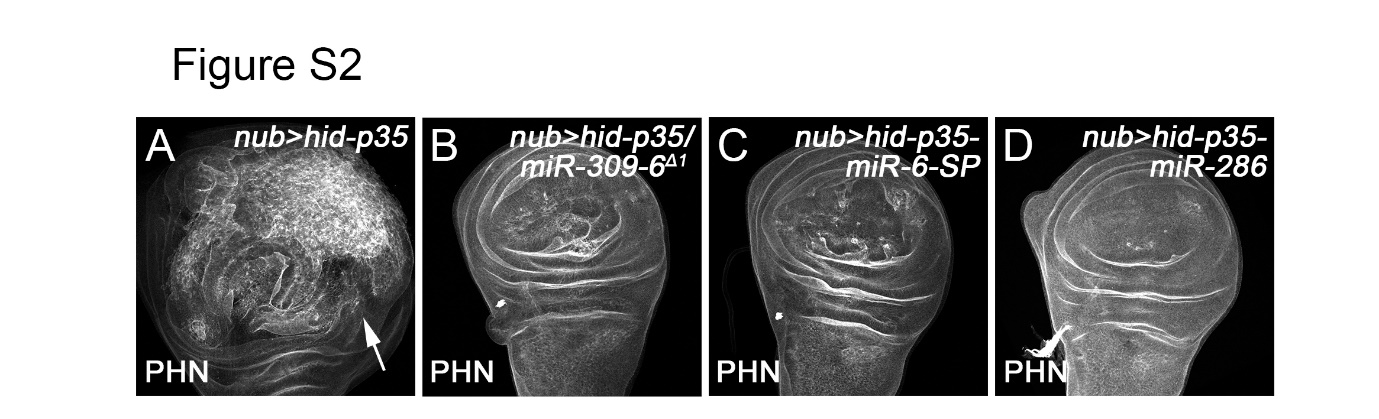


**Supplemental Figure S2. miR-6 and miR-286 regulate AiP in developing wing discs.**

(A-D) Late 3^rd^ instar wing discs labelled with Phalloidin (PHN), a marker of F-actin. *nub>hid-p35* discs show tissue overgrowth characterized by enlarged wing pouch and a massive increase of F-actin filaments (arrow, A). This tissue overgrowth is suppressed by heterozygous *miR-309-6^Δ1^* (B) or expression of *miR-6-SP* (C) or miR-286 overexpression (D).


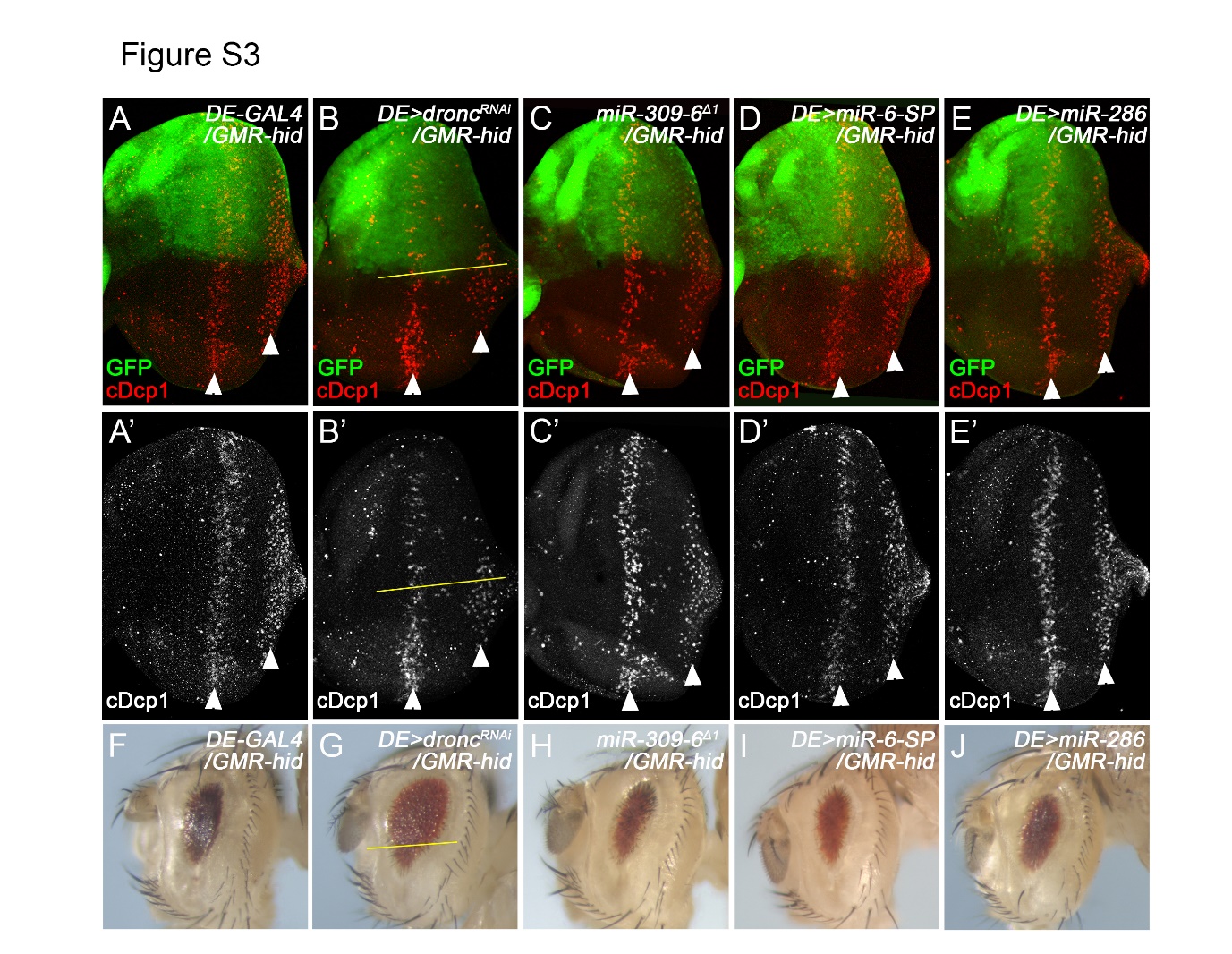


**Supplemental Figure S3. Expression of *miR-6-SP* or miR-286 does not inhibit *hid*-induced apoptosis.**

(A-E) Late 3^rd^ instar eye discs expressing a *GMR-hid* transgene to induce apoptosis, anterior is to the left. (A’-E’) are single channel images of (A-E). *DE-GAL4* was used to drive expression of various *UAS*-constructs in these discs. The cleaved Dcp-1 (cDcp-1) antibodies label apoptotic cells. *GMR-hid* induces two waves of apoptosis as shown in the control (A, arrowheads). Expression of *dronc^RNAi^* under the control of *DE-GAL4* suppresses apoptosis in the dorsal half of the disc (B, highlighted by the yellow line). In contrast, heterozygous *miR-309-6^Δ1^* (C) or expression of *miR-6-SP* (D) or miR-286 (E) does not affect *GMR-hid*-induced apoptosis.

(F-J) Representative adult fly eye images of the indicated genotypes. *GMR-hid* induces an eye ablation phenotype as shown in the control (F). Expression of *dronc^RNAi^* driven by *DE-GAL4* partially rescues the dorsal part of the eye due to its suppression of *GMR-hid*-induced apoptosis in this region (G, highlighted by the yellow line). However, heterozygous *miR-309-6^Δ1^* (H) or expression of *miR-6-SP* (I) or miR-286 (J) does not affect *GMR-hid*-induced eye ablation phenotype.

| **Table S1. Predicted gene targets of miR-286 by TargetScanFly, PicTar and miRanda** | | | |
| --- | --- | --- | --- |
| TargetScanFly (miR-286-5p) | TargetScanFly (miR-286-3p) | PicTar | miRanda |
| Rab10 | nerfin-1 | nerfin-1 | neur |
| CG33774 | Mgat2 | insb | Pax |
| CG46320 | onecut | SP555 | Prosbeta7 |
| CG5958 | insb | Mgat2 | elav |
| CG13231 | robo1 | gogo | Wsck |
| Lst | fd3F | Vha68-1 | Atg13 |
| COX7A | SP555 | BORCS6 | wnd |
| CG9129 | CG14042 | INPP5E | CG5909 |
| Cpr5C | scrt | Sas-6 | CG15211 |
| RpII15 | CG9581 | CG11760 | CG3687 |
| Tapdelta | rempA | ewg | T48 |
| CG2127 | nes | boss | wdp |
| CG3226 | Appl | robo1 | Sh |
| CG6432 | tap | CG9581 | loqs |
| CG13585 | Synj | sug | CG32645 |
| ewg | msi | Cpr78Cc | Scs-fp |
| ImpL3 | CG13398 | CG9626 | Mtl |
| CG12520 | CG32532 | Root | sol |
| Sc2 | CanA1 | PMP34 | CG2658 |
| Cpr92F | Oli | CG10561 | Pi3K59F |
| CG30412 | dsb | CG4000 | SP555 |
| CG4927 | if | obst-E | CG10731 |
| CG33110 | cv-c | CG8298 | stnB |
| CG42837 | CG3940 | CG8206 | Ca-P60A |
| CG33514 | Pde6 | HPS1 | Cyp4g1 |
| CG13028 | elav | Hey | Rya-r44F |
| CG9662 | CG12024 | CG45064 | CG9339 |
| yin | CG13917 | CG15212 | CG7896 |
| CG5447 | geko | CAH7 | CG7375 |
| pre-mod(mdg4)-AD | CG7745 | Oli | Tsp39D |
| side | CG13124 | CG11617 | Abl |
| CG32298 | cwo | inv | CG41106 |
| Syx16 | Ntmt | CG9083 | CG3016 |
| TER94 | Arfip | PIG-F | mus312 |
| Lcp65Ad | rho | sc | CG13284 |
| AdSL | Sur-8 | CG15096 | ND42 |
| NO66 | CG9626 | CanA1 | CG15814 |
| CG5608 | Abl | Abl | CG14408 |
| Adgf-A | Hey | geko | CG11198 |
| CG31538 | CG13255 | CG2061 | CG8298 |
| SH3PX1 | CG1504 | CG32532 | Pld |
| CG9368 | upd1 | RyR | Rbp9 |
| Rap1 | Toll-9 | nes | SelR |
| ZnT33D | CG8298 | DCP2 | obst-E |
| fal | dtn | sol | CG11760 |
| ninaB | Hr38 | Rgk1 | Appl |
| Drip | gogo | Usp8 | CG7785 |
| CG12963 | Gbs-76A | Sh | fz2 |
| CG8500 | boss | G9a | onecut |
| CG30380 | neo | ktub | SIFR |
| CG10948 | Pvf3 | twe | CG11883 |
| pre-mod(mdg4)-AE | ps | CG8272 | CG32813 |
| CG4282 | CG2061 | ru | HP4 |
| CG11381 | sky | Rbp9 | pall |
| NaCP60E | Hip14 | CG13398 | CG17184 |
| dah | ex | Usp30 | Mkk4 |
| CG7606 | CG43340 | CG14408 | CaMKI |
| CG42577 | CG13300 | T48 | CG6114 |
| CtsB1 | axed | exex | Mpcp |
| CG7888 | CG12054 | Dhit | bcd |
| Vha36-1 | Dys | Uxs | Syn |
| CG13000 | CG15814 | lab | CG1637 |
| CG30286 | x16 | CG15814 | D |
| CG14579 | bru3 | Mtl | gogo |
| Chd64 | sc | fus | CG1962 |
| CG11444 | Mtl | att-ORFB | Hrb98DE |
| CG30158 | isopeptidase-T-3 | Arfip | twe |
| lmgA | PIG-F | Naa80 | CG10512 |
| CG34196 | CG18265 | CG43341 | ase |
| Fer1HCH | CG13344 | Dab | pod1 |
| tll | Gyc76C | cwo | Rat1 |
| CG15892 | G9a | Ac78C | sls |
| trus | CG1172 | CG6752 | CG6310 |
| Mec2 | CG13731 | BTBD9 | Plc21C |
| CG15482 | Awh | gcm | CAP |
| brp | Pkc98E | nvy | exex |
| Or43a | CG4000 | esg | CG11906 |
| CG6218 | CG33978 | CG42637 | RpL13A |
| dpr6 | CG2217 | Awh | boss |
| pre-mod(mdg4)-X | Samuel | upd1 | CG7802 |
| CG5885 | CG43739 | tap | CG34224 |
| CG8839 | CG15097 | isopeptidase-T-3 | ewg |
| Oamb | brat | CAP | inv |
| Dlg5 | neur | x16 | CG3714 |
| spict | sug | CG13344 | spin |
| Ssrp | Ncoa6 | fng | opa1-like |
| CG11180 | CG16791 | CG15097 | CG14292 |
| CG13244 | DIP-alpha | Ptx1 | mus81 |
| CG5359 | ChAT | Synj | nudE |
| CG15096 | CAP | Trl | CG12605 |
| sun | INPP5E | Tie | Atet |
| CG10880 | Uxs | rho | CG13344 |
| CG31075 | gcm | CG12024 | CG34178 |
| Surf4 | CG13004 | CG32813 | Nc73EF |
| baf | CG14440 | fz2 | CG7255 |
| Sry-alpha | CG8481 | Calx | CG15097 |
| Vha44 | NetB | cv-c | Rb97D |
| Set | CG14408 | if | CG3967 |
| Notum | gol | neur | CG13908 |
| CG46319 | BTBD9 | Cul5 | Vha44 |
| CG15537 | tamo | CG6282 | CG31498 |
| CG3198 | Pph13 | ex | CG31635 |
| CG3655 | ITP | Pkc98E | CG6282 |
| CAH1 | Zasp66 | CG2519 | CG11253 |
| goe | CG11617 | milt | sas-6 |
| CG5762 | inv | tamo | CG4004 |
| CG42578 | CG11221 | Pde6 | CG17129 |
| Mvb12 | Cht6 | CG6175 | CG3542 |
| AMPdeam | ewg | neo | CG9467 |
| CG6813 | CG45065 | Gbs-76A | pie |
| CG2781 | CG45064 | CG18265 | Calx |
| CG11629 | CG46306 | Ncoa6 | CG17352 |
| para | Dab | B4 | CG10137 |
| Rim | CG10019 | Sur-8 | CG32048 |
| cmpy | l(3)neo38 | ss | CG12428 |
| rl | Kah | sky | CG10561 |
| CG43815 | Calx | Pif1B | CG8213 |
| Ca-beta | CG11760 | Tollo | CG30088 |
| fok | TMEM216 | Hr38 | CG9517 |
| Osi7 | CG2225 | NetB | Zpr1 |
| Mco1 | Fer1 |  | CG6995 |
| CG1677 | mbf1 |  | CG34450 |
| CG13511 | GlcT-1 |  | rdgC |
| CG11550 | CG43078 |  | Atg7 |
| ssp3 | CG6175 |  | Drep-2 |
| CG5439 | ru |  | CG10151 |
| Wsck | CG42637 |  | vib |
| CG42488 | nAChRalpha3 |  | Dcp2 |
| Stip1 | ect |  | CG17019 |
| Vps53 | CG10561 |  | CG12734 |
| CG3065 | Tollo |  | emp |
| CG42307 | Mp |  | CG6428 |
| Dredd | CG32813 |  | Dgp-1 |
| CG34210 | CG2865 |  | CBP |
| CG12594 | RunxA |  | tai |
| fh | exex |  | CG4101 |
| CG4465 | fng |  | vlc |
| exex | Osi7 |  | Cad87A |
| nahoda | T48 |  | CG18549 |
| CG32631 | CG10738 |  | H2.0 |
| Ero1L | Rfx |  | Sur |
| Cortactin | lab |  | cv-c |
| grnd | CaMKI |  | dsx |
| Pgam5-2 | Dhit |  | CG13255 |
| CG8170 | Sp1 |  | tamo |
| Ubc4 | CG15212 |  | CG6767 |
| fl(2)d | CG9083 |  | CG6151 |
| CG11029 | ss |  | lola |
| Mes4 | PTP-ER |  | achi |
| pre-mod(mdg4)-Y | CG15096 |  | CG17193 |
| Gr64b | tnc |  | CG6041 |
| CG31010 | Usp8 |  | lilli |
| CG9149 | Pka-R2 |  | Mgat2 |
| h | Ptx1 |  | TwdlE |
| CG32301 | Mur18B |  | CG15212 |
| CG17207 | CG5027 |  | Sur-8 |
| CG16717 | obst-E |  | obst-A |
| Lkb1 | Ac78C |  | bs |
| Arc1 | esg |  | CG32112 |
| scaf6 | milt |  | bsh |
| Ppn | CG7785 |  | CG3523 |
| Glut1 | CG34007 |  | CG2964 |
| CG31324 | Tie |  | ex |
| heix | CG6428 |  | CG17180 |
| CG7720 | CG16779 |  | CG4467 |
| CG14985 | CG8500 |  | CG4662 |
| GLS | Rbp6 |  | L |
| mle | Rgk1 |  | CG42637 |
| Cht7 | CG32767 |  | l(2)01810 |
| mv | fz2 |  | os |
| Ubc2 | CG2519 |  | CG13995 |
| CG31998 | B4 |  | CG11617 |
| CG33095 | HPS1 |  | cort |
| shakB | RyR |  | CG7971 |
| CG30177 | DCP2 |  | CG9581 |
| kek5 | fus |  | CG8850 |
| rad | CG6752 |  | fus |
| hoe2 | bsh |  | CG8317 |
| CG4962 | Atg7 |  | beat-IIa |
| CG45085 | Nrk |  | CG1213 |
| Vps2 | twe |  | ninaC |
| CG2162 | ktub |  | CG4089 |
| Calx | mxt |  | CG17181 |
| Nop60B | Rbp9 |  | CG2061 |
| CG10845 | CG32333 |  | frtz |
| Spn42Db | ste14 |  | ps |
| CG11380 | Trpm |  | dl |
| Cp38 | pHCl-1 |  | GluClalpha |
| CG42542 | CG5890 |  | smg |
| CNT1 | CG6282 |  | CG7058 |
| CG8974 | pod1 |  | CG5690 |
| pav | CG11034 |  | CG12187 |
| CG32581 | CG14505 |  | CG15270 |
| CG10494 | hang |  | Awh |
| eEF1alpha2 | CG12768 |  | Vha68-1 |
| amon | bcd |  | CG8964 |
| CG13606 | haf |  | CG8677 |
| CG11723 | Cpr78Cc |  | Cha |
| CG42340 | CG4676 |  | Mip |
| lin-52 | Cht2 |  | cul-5 |
| CG7872 | Kdm2 |  | Ptx1 |
| CG7886 | CG17180 |  | osk |
| CG7991 | Vha68-1 |  | CG9018 |
| exu | Elk |  | CG13124 |
| BI-1 | CG1402 |  | CG14570 |
| inaF-B | Lap1 |  | Act88F |
| vis | Pkcdelta |  | Hk |
| spidey | CG31064 |  | CG10077 |
| Gr61a | Fife |  | CG8155 |
| Fem-1 | Saf-B |  | Hey |
| RhoGAP15B | Sas-6 |  | CLIP-190 |
| CG33523 | nvy |  | Trl |
| CG30424 | Pvf2 |  | DopR2 |
| Syx7 | CG8272 |  | Pph13 |
| CG1402 | hep |  | Best2 |
| yem | tinc |  | HPS |
| CG10344 | CG34383 |  | Gyc88E |
| Task6 | P5CDh1 |  | CG10566 |
| ced-6 |  |  | Mgstl |
| BuGZ |  |  | CG4238 |
| CG8176 |  |  | Arp53D |
| LIMK1 |  |  | Mur18B |
| CG9766 |  |  | ste14 |
| CG34404 |  |  | CG14322 |
| ken |  |  | CG14869 |
| Set2 |  |  | ru |
| pgc |  |  | CG14023 |
| Rchy1 |  |  | CcapR |
| CG8745 |  |  | CG9265 |
| CG13055 |  |  | y |
| CG10979 |  |  | gcm |
| beat-VI |  |  | Adgf-D |
| CG45075 |  |  | Tsp66A |
| CG33096 |  |  | CG13675 |
| CG8918 |  |  | CG11155 |
| CG7115 |  |  | CG11454 |
| CG6282 |  |  | CG31646 |
| inaF-C |  |  | Cnx99A |
| CG9008 |  |  | Hr38 |
| DOR |  |  | CG11168 |
| achi |  |  | alpha-catenin-related |
| CG46244 |  |  | king-tubby |
| pAbp |  |  | CG18011 |
| dock |  |  | CG9760 |
| CycA |  |  | CG5798 |
| CG42594 |  |  | milt |
| CG13579 |  |  | CG1789 |
| inaF-A |  |  | Pkcdelta |
| CG34345 |  |  | dap |
| Drep2 |  |  | B4 |
| CG10413 |  |  | TwdlBeta |
| Ubc6 |  |  | dpr18 |
| SP555 |  |  | isopeptidase-T-3 |
| Pdi |  |  | Dgkepsilon |
| GSS |  |  | S2P |
| CG32495 |  |  | m-cup |
| CG34293 |  |  | tin |
| Nmdar2 |  |  | synj |
| CG10420 |  |  | inaE |
| CG42556 |  |  | Dap160 |
| Act5C |  |  | CG8931 |
| FucTA |  |  | CG18031 |
| ergic53 |  |  | CG13333 |
| MESR4 |  |  | tinc |
| rb |  |  | CG7927 |
| CG11897 |  |  | eIF4E-7 |
| CG42516 |  |  | Jon25Bi |
| Hip14 |  |  | CG30190 |
| Or59b |  |  | CG3862 |
| Or10a |  |  | hang |
| CG13127 |  |  | sinah |
| hoe1 |  |  | CG31064 |
| Cyt-b5 |  |  | Rgk1 |
| TM9SF3 |  |  | wit |
| CG4538 |  |  | Gasp |
| X11L |  |  | CG34441 |
| tap |  |  | svp |
| CG7326 |  |  | CG32564 |
| Mbs |  |  | CG4313 |
| smog |  |  | esg |
| Reep1 |  |  | cni |
| Ca-alpha1D |  |  | nerfin-1 |
| Fbl6 |  |  | puc |
| CG18675 |  |  | Trc8 |
| CG11023 |  |  | CG7101 |
| pnut |  |  | CG6734 |
| Drl-2 |  |  | cag |
| CG1909 |  |  | CG2217 |
| CG43897 |  |  | Npc2h |
| Nedd8 |  |  | CG11360 |
| CG32192 |  |  | CG14042 |
| CG32266 |  |  | SPR |
| CG2321 |  |  | CG7407 |
| CG12772 |  |  | CG4942 |
| CG46311 |  |  | CG5910 |
| Eps-15 |  |  | sens-2 |
| CG9095 |  |  | CG34203 |
| Cpr49Ac |  |  | CG30172 |
| CG33125 |  |  | bbx |
| Edem2 |  |  | trh |
| CG10103 |  |  | CG5630 |
| lili |  |  | CanA1 |
| CG15141 |  |  | lab |
| CG42661 |  |  | CG4000 |
| foxo |  |  | CG5036 |
| hts |  |  | CG15412 |
| Rbm13 |  |  | rib |
| CG30058 |  |  | CG31221 |
| CG3295 |  |  | xl6 |
| Pvf3 |  |  | robo |
| CG9514 |  |  | Arf72A |
| CG6978 |  |  | CG1826 |
| gogo |  |  | CG14210 |
| CG42673 |  |  | Ac78C |
| CG11448 |  |  | rost |
| wgn |  |  | SerT |
| MsR1 |  |  | Dab |
| eyg |  |  | CG9619 |
| DIP-theta |  |  | CG5087 |
| CG9990 |  |  | CG5327 |
| Hk |  |  | CG10300 |
| fdl |  |  | unc-5 |
| LRR |  |  | CG13188 |
| Culd |  |  | CG14505 |
| htl |  |  | ss |
| pns |  |  | CG11035 |
| CG8475 |  |  | CG3940 |
| Liprin-beta |  |  | CG5027 |
| ctp |  |  | Pkc98E |
| CG43861 |  |  | CG2519 |
| CG46335 |  |  | fng |
| hubl |  |  | robl62A |
| CG9813 |  |  | Snoo |
| Duba |  |  | msl-3 |
| CG12290 |  |  | CG6520 |
| tipE |  |  | CG7979 |
| AstA-R1 |  |  | Fas2 |
| sv |  |  | run |
| Mkk4 |  |  | CG2104 |
| CG34235 |  |  | CG18265 |
| godzilla |  |  | Tie |
| LRP1 |  |  | CG8500 |
| sing |  |  | PGRP-LD |
| Lerp |  |  | stwl |
| Hdc |  |  | CG18649 |
| fu |  |  | Proct |
| RluA-1 |  |  | CG9222 |
| Pde11 |  |  | nvy |
| CG14082 |  |  | CG32418 |
| CG7370 |  |  | Ugt86Dd |
| dbo |  |  | prel |
| vih |  |  | sisA |
| Scamp |  |  | sug |
| CG3638 |  |  | att-ORFA |
| CG17271 |  |  | Su(H) |
| Snapin |  |  | CG8206 |
| CG31638 |  |  | CG12177 |
| CG42660 |  |  | CG9376 |
| Cdk12 |  |  | Pp1-13C |
| Gfrl |  |  | CG11317 |
| PCB |  |  | Fmrf |
| CG34447 |  |  | CG2016 |
| ari-2 |  |  | corn |
| Pli |  |  | CG11741 |
| CG42613 |  |  | Pbgs |
| cal1 |  |  | CG13481 |
| CG31465 |  |  | CG9083 |
| CG46312 |  |  | CG34370 |
| CG3689 |  |  | CG33960 |
| alpha-Man-Ib |  |  | npf |
| zfh1 |  |  | G9a |
| ChAT |  |  | CG3689 |
| Madm |  |  | CG18476 |
| Mapmodulin |  |  | CG5053 |
| Tsp5D |  |  | CG6752 |
| CG14762 |  |  | CG9626 |
| Ada3 |  |  | Syt12 |
| Rat1 |  |  | Psf3 |
| inaF-D |  |  | wus |
| ps |  |  | Tsp26A |
| CG3548 |  |  | Pat1 |
| Vha26 |  |  | Samuel |
| AspRS-m |  |  | VGlut |
| CG13300 |  |  | CG13154 |
| Tm2 |  |  | CG6073 |
| CG46315 |  |  | CG32250 |
| oys |  |  | CG4439 |
| nAChRbeta2 |  |  | CG18335 |
| ITP |  |  | sc |
| beat-IIIa |  |  | CG8272 |
| Octbeta2R |  |  | ect |
| pgant8 |  |  |  |
| CG30343 |  |  |  |
| Upf1 |  |  |  |
| jp |  |  |  |
| CG13685 |  |  |  |
| Prosap |  |  |  |
| CG6425 |  |  |  |
| CG14042 |  |  |  |
| caps |  |  |  |
| CG8602 |  |  |  |
| Xe7 |  |  |  |
| yata |  |  |  |
| dpr11 |  |  |  |
| Piezo |  |  |  |
| eIF3e |  |  |  |
| TfIIB |  |  |  |
| Tpc1 |  |  |  |
| CG13931 |  |  |  |
| mus312 |  |  |  |
| pyd |  |  |  |
| CG3009 |  |  |  |
| CG6424 |  |  |  |
| pasha |  |  |  |
| CG31542 |  |  |  |
| jeb |  |  |  |
| CG11168 |  |  |  |
| Indy-2 |  |  |  |
| Ran |  |  |  |
| Myo95E |  |  |  |
| CG10960 |  |  |  |
| Gnmt |  |  |  |
| mGluR |  |  |  |
| CG4297 |  |  |  |
| l(2)01289 |  |  |  |
| CG32576 |  |  |  |
| beat-Vc |  |  |  |
| dikar |  |  |  |
| CG44477 |  |  |  |
| CG7290 |  |  |  |
| stck |  |  |  |
| Grd |  |  |  |
| Mhc |  |  |  |
| spir |  |  |  |
| Dyb |  |  |  |
| CG6406 |  |  |  |
| Syn2 |  |  |  |
| CG4022 |  |  |  |
| Sry-beta |  |  |  |
| KdelR |  |  |  |
| CG13284 |  |  |  |
| CG8005 |  |  |  |
| Pcyt2 |  |  |  |
| Abd-B |  |  |  |
| CG6136 |  |  |  |
| CG8726 |  |  |  |
| CG2685 |  |  |  |
| sd |  |  |  |
| CG7414 |  |  |  |
| CG45002 |  |  |  |
| CG42512 |  |  |  |
| CG4238 |  |  |  |
| CG14459 |  |  |  |
| qless |  |  |  |
| mahe |  |  |  |
| CG14435 |  |  |  |
| CG13510 |  |  |  |
| Proc-R |  |  |  |
| CG12769 |  |  |  |
| Elk |  |  |  |
| H |  |  |  |
| CG5937 |  |  |  |
| CG17528 |  |  |  |
| CG13023 |  |  |  |
| CG7139 |  |  |  |
| rhea |  |  |  |
| CG14372 |  |  |  |
| Cralbp |  |  |  |
| CG9674 |  |  |  |
| CG1360 |  |  |  |
| CG1983 |  |  |  |
| sff |  |  |  |
| CG45086 |  |  |  |
| rg |  |  |  |
| CG15465 |  |  |  |
| M6 |  |  |  |
| RunxA |  |  |  |
| tutl |  |  |  |
| UbcE2M |  |  |  |
| Eip78C |  |  |  |
| Scr |  |  |  |
| faf |  |  |  |
| Dop1R1 |  |  |  |
| CG4928 |  |  |  |
| CG6928 |  |  |  |
| 312 |  |  |  |
| CG12814 |  |  |  |
| Pak3 |  |  |  |
| CG34377 |  |  |  |
| CG5674 |  |  |  |
| CG17786 |  |  |  |
| CG6123 |  |  |  |
| CG12605 |  |  |  |
| CG43110 |  |  |  |
| sm |  |  |  |
| sl |  |  |  |
| CG6118 |  |  |  |
| dpr1 |  |  |  |
| ND-B12 |  |  |  |
| trio |  |  |  |
| sgg |  |  |  |
| Mical |  |  |  |
| SK |  |  |  |
| Rab3-GEF |  |  |  |
| pros |  |  |  |
| flw |  |  |  |
| CG42265 |  |  |  |
| ens |  |  |  |
| RacGAP84C |  |  |  |
| CG45087 |  |  |  |
| pan |  |  |  |
| Vps28 |  |  |  |
| nmo |  |  |  |
| TwdlV |  |  |  |
| CG34383 |  |  |  |
| CG6527 |  |  |  |
| Rbp |  |  |  |
| CG11505 |  |  |  |
| CG1421 |  |  |  |
| SKIP |  |  |  |
| Usp1 |  |  |  |
| Ir40a |  |  |  |
| CG12659 |  |  |  |
| CG7556 |  |  |  |
| fru |  |  |  |
| CG4587 |  |  |  |
| nej |  |  |  |
| Cp7Fb |  |  |  |
| nAChRalpha6 |  |  |  |
| galla-2 |  |  |  |
| yellow-e2 |  |  |  |
| Teh1 |  |  |  |
| Tbh |  |  |  |
| RhoGAP102A |  |  |  |
| okr |  |  |  |
| chrb |  |  |  |
| CG45101 |  |  |  |
| cno |  |  |  |
| Tyler |  |  |  |
| trp |  |  |  |
| CG13506 |  |  |  |
| ena |  |  |  |
| CG8213 |  |  |  |
| CG17715 |  |  |  |
| bru2 |  |  |  |
| beat-Ia |  |  |  |
| CG42534 |  |  |  |
| nwk |  |  |  |
| Snup |  |  |  |
| ClC-a |  |  |  |
| CG44422 |  |  |  |
| Zn72D |  |  |  |
| CG43367 |  |  |  |
| Octbeta3R |  |  |  |
| DIP-delta |  |  |  |
| NimB1 |  |  |  |
| Asph |  |  |  |
| Synj |  |  |  |
| THADA |  |  |  |
| scyl |  |  |  |
| CIAPIN1 |  |  |  |
| hdly |  |  |  |
| Fatp |  |  |  |
| slmo |  |  |  |
| Syt7 |  |  |  |
| CG17162 |  |  |  |
| Arfrp1 |  |  |  |
| Amy-p |  |  |  |
| prominin-like |  |  |  |
| Frq1 |  |  |  |
| CG31323 |  |  |  |
| CG44195 |  |  |  |
| Dlic |  |  |  |
| CG43373 |  |  |  |
| ldbr |  |  |  |
| CG43689 |  |  |  |
| ssp6 |  |  |  |
| Fas1 |  |  |  |
| CG32698 |  |  |  |
| CG17159 |  |  |  |
| tzn |  |  |  |
| CG4004 |  |  |  |
| CG10924 |  |  |  |
| HSPC300 |  |  |  |
| CG40498 |  |  |  |
| CG31710 |  |  |  |
| DIP-alpha |  |  |  |
| lqf |  |  |  |
| RanBPM |  |  |  |
| Nckx30C |  |  |  |
| Hex-A |  |  |  |
| tst |  |  |  |
| 14-3-3epsilon |  |  |  |
| CG14269 |  |  |  |
| Gmap |  |  |  |
| Camta |  |  |  |
| rdgA |  |  |  |
| Pink1 |  |  |  |
| CG9698 |  |  |  |
| sol |  |  |  |
| didum |  |  |  |
| CG2217 |  |  |  |
| nAChRalpha3 |  |  |  |
| sli |  |  |  |
| scrib |  |  |  |
| CG13175 |  |  |  |
| CG17075 |  |  |  |
| scrt |  |  |  |
| RhoGAP18B |  |  |  |
| Gbs-76A |  |  |  |
| Mur2B |  |  |  |
| CG12912 |  |  |  |
| Sf3b3 |  |  |  |
| Su(var)3-7 |  |  |  |
| Eip74EF |  |  |  |
| CG46321 |  |  |  |
| grn |  |  |  |
| ths |  |  |  |
| pum |  |  |  |
| Not11 |  |  |  |
| mgl |  |  |  |
| lwr |  |  |  |
| CG6154 |  |  |  |
| NKAIN |  |  |  |
| CG11665 |  |  |  |
| kuz |  |  |  |
| eff |  |  |  |
| CG6966 |  |  |  |
| Asciz |  |  |  |
| CG8079 |  |  |  |
| RhoGAP19D |  |  |  |
| CG9886 |  |  |  |
| beat-VII |  |  |  |
| Octbeta1R |  |  |  |
| tou |  |  |  |
| Alk |  |  |  |
| cm |  |  |  |
| RpL12 |  |  |  |
| CG34357 |  |  |  |
| fs(1)h |  |  |  |
| elav |  |  |  |
| SmD3 |  |  |  |
| Top1 |  |  |  |
| CG3262 |  |  |  |
| CG43902 |  |  |  |
| CG7510 |  |  |  |
| Rdl |  |  |  |
| CG7601 |  |  |  |
| CG14823 |  |  |  |
| CG44434 |  |  |  |
| gish |  |  |  |
| r2d2 |  |  |  |
| Ank2 |  |  |  |
| nsl1 |  |  |  |
| Naa15-16 |  |  |  |
| CG1090 |  |  |  |
| vib |  |  |  |
| Lis-1 |  |  |  |
| Smr |  |  |  |
| Pkc98E |  |  |  |
| CG13887 |  |  |  |
| CG16964 |  |  |  |
| Dim1 |  |  |  |
| mRpL17 |  |  |  |
| CG11109 |  |  |  |
| gdl |  |  |  |
| CG10466 |  |  |  |
| CG14402 |  |  |  |
| Idgf3 |  |  |  |
| CG6805 |  |  |  |
| CG7309 |  |  |  |
| Imp |  |  |  |
| lilli |  |  |  |
| Grip84 |  |  |  |
| Rbp6 |  |  |  |
| Spn |  |  |  |
| CG9399 |  |  |  |
| hdc |  |  |  |
| CG17684 |  |  |  |
| mtd |  |  |  |
| CG1418 |  |  |  |
| Cyp312a1 |  |  |  |
| CG9175 |  |  |  |
| CG11138 |  |  |  |
| stau |  |  |  |
| CG32447 |  |  |  |
| mus301 |  |  |  |
| CG4570 |  |  |  |
| Rbp9 |  |  |  |
| zld |  |  |  |
| thoc6 |  |  |  |
| CG15676 |  |  |  |
| CG31709 |  |  |  |
| dnc |  |  |  |
| CG12831 |  |  |  |
| Ilp6 |  |  |  |
| retm |  |  |  |
| Eip93F |  |  |  |
| Cyp6a18 |  |  |  |
| uzip |  |  |  |
| Btk29A |  |  |  |
| Spn47C |  |  |  |
| Shab |  |  |  |
| CG5280 |  |  |  |
| Ucp4B |  |  |  |
| Sfp77F |  |  |  |
| CG11906 |  |  |  |
| CG3163 |  |  |  |
| CG7324 |  |  |  |
| otp |  |  |  |
| gpp |  |  |  |
| scra |  |  |  |
| 140up |  |  |  |
| CG1958 |  |  |  |
| sut1 |  |  |  |
| CG6073 |  |  |  |
| Tao |  |  |  |
| cic |  |  |  |
| ple |  |  |  |
| Gp210 |  |  |  |
| jet |  |  |  |
| CG6379 |  |  |  |
| CG44325 |  |  |  |
| Usp14 |  |  |  |
| CG5639 |  |  |  |
| CG2493 |  |  |  |
| Mlp84B |  |  |  |
| CG12851 |  |  |  |
| Unr |  |  |  |
| Hrb27C |  |  |  |
| Obp51a |  |  |  |
| l(2)37Cc |  |  |  |
| CG45263 |  |  |  |
| Atf6 |  |  |  |
| CG5597 |  |  |  |
| Sh |  |  |  |
| CG1172 |  |  |  |
| CG6726 |  |  |  |
| CG14408 |  |  |  |
| wde |  |  |  |
| robo3 |  |  |  |
| Tango6 |  |  |  |
| CG8526 |  |  |  |
| CCT2 |  |  |  |
| Mf |  |  |  |
| Myc |  |  |  |
| FANCI |  |  |  |
| Df31 |  |  |  |
| vir-1 |  |  |  |
| CG15208 |  |  |  |
| orb2 |  |  |  |
| GABA-B-R3 |  |  |  |
| Parp |  |  |  |
| CG17598 |  |  |  |
| CG3337 |  |  |  |
| MRP |  |  |  |
| nAChRalpha5 |  |  |  |
| Actn |  |  |  |
| rst |  |  |  |
| Ac13E |  |  |  |
| CG33506 |  |  |  |
| m-cup |  |  |  |
| CG4768 |  |  |  |
| CG7627 |  |  |  |

| **Table S2. Overlapping miR-286 targets predicted by TargetScanFly, PicTar and miRanda** | | |
| --- | --- | --- |
| TargetScanFly (miR-286-5p) *vs* PicTar *vs* miRanda | TargetScanFly (miR-286-3p) *vs* PicTar *vs* miRanda | Overlapping Targets |
| Calx | Abl | Calx |
| CG14408 | Ac78C | CG14408 |
| CG6282 | Awh | CG6282 |
| ewg | B4 | ewg |
| exex | boss | exex |
| gogo | Calx | gogo |
| Pkc98E | CanA1 | Pkc98E |
| Rbp9 | CAP | Rbp9 |
| Sh | CG10561 | SP555 |
| sol | CG11617 | Synj |
| SP555 | CG11760 |  |
| Synj | CG13344 |  |
|  | CG14408 |  |
|  | CG15097 |  |
|  | CG15212 |  |
|  | CG15814 |  |
|  | CG18265 |  |
|  | CG2061 |  |
|  | CG2519 |  |
|  | CG32813 |  |
|  | CG4000 |  |
|  | CG42637 |  |
|  | CG6282 |  |
|  | CG6752 |  |
|  | CG8272 |  |
|  | CG8298 |  |
|  | CG9083 |  |
|  | CG9581 |  |
|  | CG9626 |  |
|  | cv-c |  |
|  | Dab |  |
|  | DCP2 |  |
|  | esg |  |
|  | ewg |  |
|  | ex |  |
|  | exex |  |
|  | fng |  |
|  | fus |  |
|  | fz2 |  |
|  | G9a |  |
|  | gcm |  |
|  | gogo |  |
|  | Hey |  |
|  | Hr38 |  |
|  | inv |  |
|  | isopeptidase-T-3 |  |
|  | lab |  |
|  | Mgat2 |  |
|  | milt |  |
|  | Mtl |  |
|  | nerfin-1 |  |
|  | neur |  |
|  | nvy |  |
|  | obst-E |  |
|  | Pkc98E |  |
|  | Ptx1 |  |
|  | Rbp9 |  |
|  | Rgk1 |  |
|  | ru |  |
|  | Sas-6 |  |
|  | sc |  |
|  | SP555 |  |
|  | ss |  |
|  | sug |  |
|  | Sur-8 |  |
|  | Synj |  |
|  | T48 |  |
|  | tamo |  |
|  | Tie |  |
|  | twe |  |
|  | Vha68-1 |  |

**Table S3. Primers used for plasmid construction and luciferase reporter assay**

| Name | Primer sequence ( 5 '- 3' ) | |
| --- | --- | --- |
| miR-6-1-3p-F  miR-1002-R  miR-137-F  miR-137-R  miR-13a-3p-F  miR-13a-3p-R  miR-184-3p-F  miR-184-3p-R  miR-2b-1-3p-F  miR-2b-1-3p-R  miR-2b-2-F  miR-2b-2-R  miR-263b-F  miR-263b-R  miR-277-3p-F  miR-277-3p-R  miR-281-2-5p-F  miR-281-2-5p-R  miR-284-F  miR-284-R  miR-3-F  miR-3-R  miR-33-5p-F  miR-33-5p-R  miR-34-F  miR-34-R  miR-375-F  miR-375-R  miR-7-F  miR-7-R  miR-932-5p-F  miR-932-5p-R  miR-966-5p-F | CGGAATTCTGACTAGACCGAACACTCGTGCT  GGTCCAGTTTTTTTTTTTTTTTAGTTAAG  GCAGTATTGCTTGAGAATACAC  GTCCAGTTTTTTTTTTTTTTTCTACG  CAGTATCACAGCCATTTTGATG  GGTCCAGTTTTTTTTTTTTTTTACTC  CAGTGGACGGAGAACTGA  CCAGTTTTTTTTTTTTTTTGCCCTT  AGTATCACAGCCAGCTTTG  GTCCAGTTTTTTTTTTTTTTTGCTC  CAGTTCTTCAAAGTGGTTGTG  GGTCCAGTTTTTTTTTTTTTTTCATTTC  GGTGGTTCTGCGGGT  TCCAGTTTTTTTTTTTTTTTGTTTTGG  GCAGTAAATGCACTATCTGGT  TCCAGTTTTTTTTTTTTTTTGTCGT  GCAGAAGAGAGCTATCCGT  GGTCCAGTTTTTTTTTTTTTTTACTGT  CCTGGAATTAAGTTGACTGTGT  TCCAGTTTTTTTTTTTTTTTGGCT  CACTGGGCAAAGTGTGT  GGTCCAGTTTTTTTTTTTTTTTGAGA  GGTGCATTGTAGTCGCA  GGTCCAGTTTTTTTTTTTTTTTGACA  GCAGTGTGGTTAGCTGGT  GTCCAGTTTTTTTTTTTTTTTCACAA  GCAGTTTGTTCGTTTGGCT  CAGGTCCAGTTTTTTTTTTTTTTTAAC  CGCAGCAATAAATCCCTTG  GTCCAGTTTTTTTTTTTTTTTAAGAAGAC  CGCAGTCAATTCCGTAGTG  CCAGTTTTTTTTTTTTTTTCTGCAATG  TGGGTTGTGGGCTGT | |
| miR-6-1-3p-R | CCCTCGAGGCAGCAGAAGTTCCCTTGGGTTA | |
| miR-6-2-3p-F  miR-1002-R  miR-137-F  miR-137-R  miR-13a-3p-F  miR-13a-3p-R  miR-184-3p-F  miR-184-3p-R  miR-2b-1-3p-F  miR-2b-1-3p-R  miR-2b-2-F  miR-2b-2-R  miR-263b-F  miR-263b-R  miR-277-3p-F  miR-277-3p-R  miR-281-2-5p-F  miR-281-2-5p-R  miR-284-F  miR-284-R  miR-3-F  miR-3-R  miR-33-5p-F  miR-33-5p-R  miR-34-F  miR-34-R  miR-375-F  miR-375-R  miR-7-F  miR-7-R  miR-932-5p-F  miR-932-5p-R  miR-966-5p-F | CGGAATTCTGCCTAACATCATTATTTAATTTTT | |
| miR-6-2-3p-R | CCCTCGAGTGAAAATGCATGTGCAAACTTAAGA | |
| miR-6-3-3p-F  miR-1002-R  miR-137-F  miR-137-R  miR-13a-3p-F  miR-13a-3p-R  miR-184-3p-F  miR-184-3p-R  miR-2b-1-3p-F  miR-2b-1-3p-R  miR-2b-2-F  miR-2b-2-R  miR-263b-F  miR-263b-R  miR-277-3p-F  miR-277-3p-R  miR-281-2-5p-F  miR-281-2-5p-R  miR-284-F  miR-284-R  miR-3-F  miR-3-R  miR-33-5p-F  miR-33-5p-R  miR-34-F  miR-34-R  miR-375-F  miR-375-R  miR-7-F  miR-7-R  miR-932-5p-F  miR-932-5p-R  miR-966-5p-F | CGGAATTCGCATTAGCAGCACCACGAGTCAA | |
| miR-6-3-3p-R | CCCTCGAGATGTCCTGGCAAACCACTGAACG | |
| miR-286-3p-F | CGGAATTCCGGTTTTGCCAATTTCCA | |
| miR-286-3p-R | CCCTCGAGTCACTTAACATAATTTCTTGACTCG | |
| miR-286-5p-F | CGGAATTCCGGTTTTGCCAATTTCCA | |
| miR-286-5p-R | CCCTCGAGTCACTTAACATAATTTCTTGACTCG | |
| miR-309-3p-F | CGGAATTCAGCCCCGACCCTTTCAGGTAACA | |
| miR-309-3p-R | CCCTCGAGGACCATACCGACATTCGCCATTC | |
| miR-309-5p-F | CGGAATTCAGCCCCGACCCTTTCAGGTAACA |  |
| miR-309-5p-R | CCCTCGAGGACCATACCGACATTCGCCATTC |  |
| *calx* 32-581 bp 3'UTR-F  miR-184-3p-F  miR-184-3p-R  miR-2b-1-3p-F  miR-2b-1-3p-R  miR-2b-2-F  miR-2b-2-R  miR-263b-F  miR-263b-R  miR-277-3p-F  miR-277-3p-R  miR-281-2-5p-F  miR-281-2-5p-R  miR-284-F  miR-284-R  miR-3-F  miR-3-R  miR-33-5p-F  miR-33-5p-R  miR-34-F  miR-34-R  miR-375-F  miR-375-R  miR-7-F  miR-7-R  miR-932-5p-F  miR-932-5p-R  miR-966-5p-F | GCTCTAGAACACGAAGCGTAAGCGTAGAGCG |  |
| *calx* 32-581 bp 3'UTR-R | GCTCTAGAGTTGCTGCGGAGTAGGGGATTTA |  |
| *calx* 1872-2401 bp 3'UTR-F  miR-184-3p-F  miR-184-3p-R  miR-2b-1-3p-F  miR-2b-1-3p-R  miR-2b-2-F  miR-2b-2-R  miR-263b-F  miR-263b-R  miR-277-3p-F  miR-277-3p-R  miR-281-2-5p-F  miR-281-2-5p-R  miR-284-F  miR-284-R  miR-3-F  miR-3-R  miR-33-5p-F  miR-33-5p-R  miR-34-F  miR-34-R  miR-375-F  miR-375-R  miR-7-F  miR-7-R  miR-932-5p-F  miR-932-5p-R  miR-966-5p-F | GCTCTAGACAACATCAAGGACTCGGTAACGG |  |
| *calx* 1872-2401 bp 3'UTR-R | GCTCTAGATTCCGCCGTAACGATCCTCTTCA |  |
| *calx* 3013-3499 bp 3'UTR-F  miR-184-3p-F  miR-184-3p-R  miR-2b-1-3p-F  miR-2b-1-3p-R  miR-2b-2-F  miR-2b-2-R  miR-263b-F  miR-263b-R  miR-277-3p-F  miR-277-3p-R  miR-281-2-5p-F  miR-281-2-5p-R  miR-284-F  miR-284-R  miR-3-F  miR-3-R  miR-33-5p-F  miR-33-5p-R  miR-34-F  miR-34-R  miR-375-F  miR-375-R  miR-7-F  miR-7-R  miR-932-5p-F  miR-932-5p-R  miR-966-5p-F | GCTCTAGACTGATTAGGGGCCTCCTTGACAA |  |
| *calx* 3013-3499 bp 3'UTR-R | GCTCTAGACGCAGTTTTCTCAACGAGTTCCC |  |
| *calx* 3042-3049 bp 3'UTR^mut^ F | CTCCTTGACAACTAGTTAGCCGCACCTCACACTATCGACA |  |
| *calx* 3042-3049 bp 3'UTR^mut^ R | TGTCGATAGTGTGAGGTGCGGCTAACTAGTTGTCAAGGAG |  |
